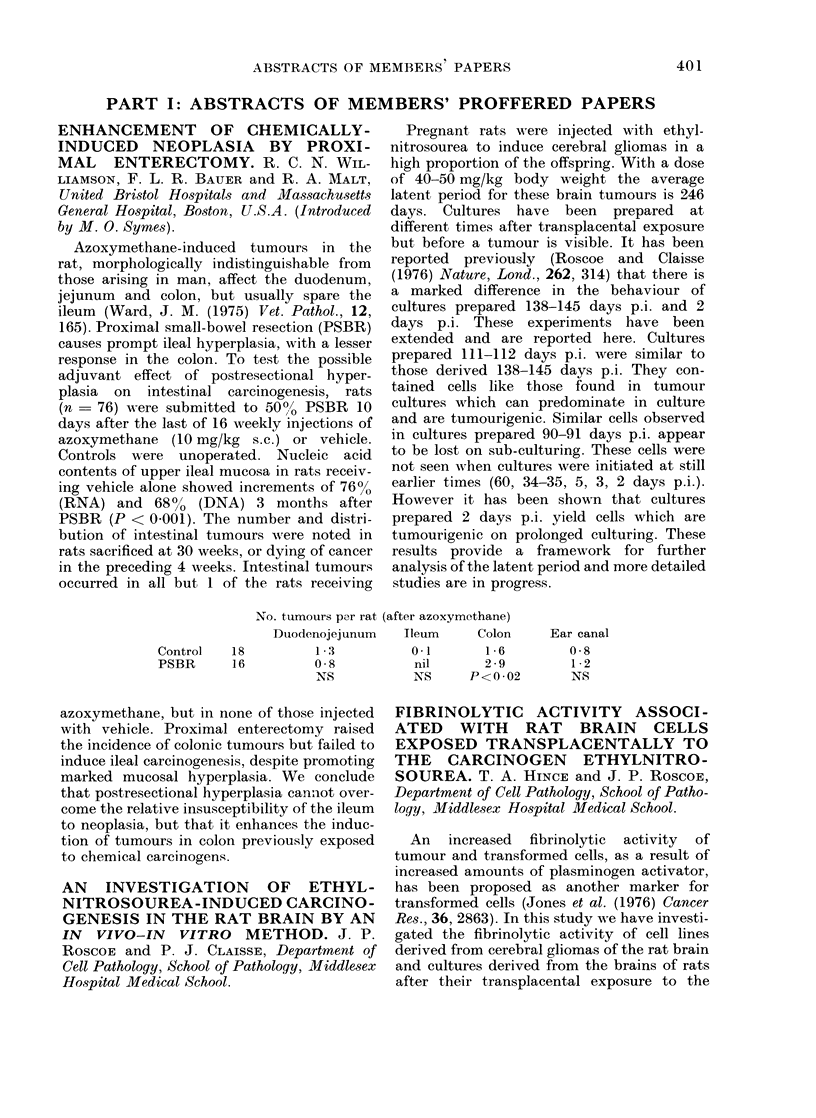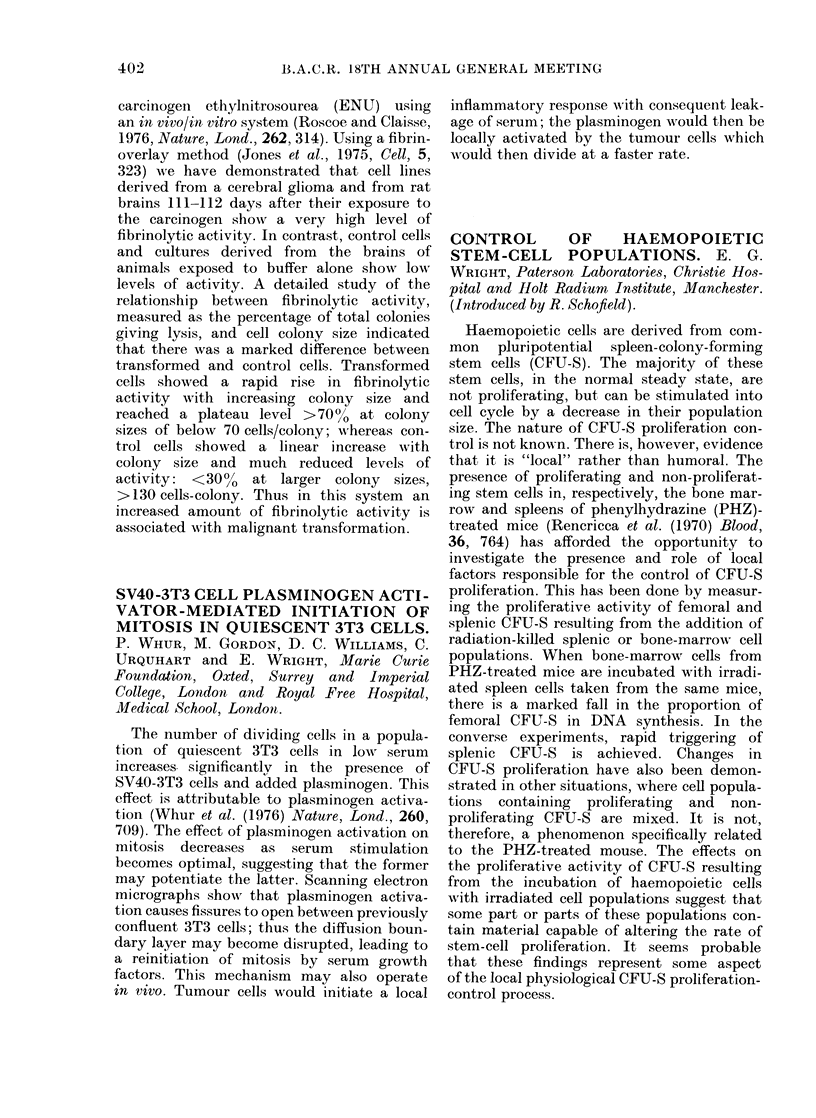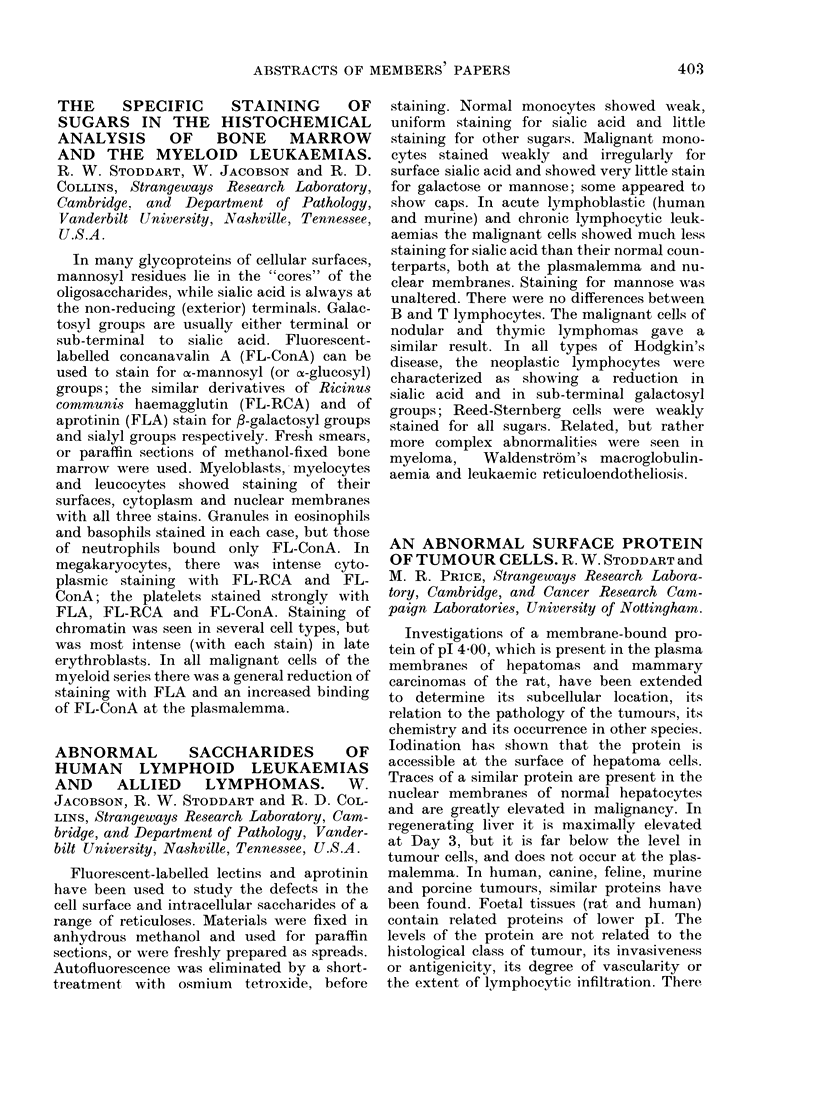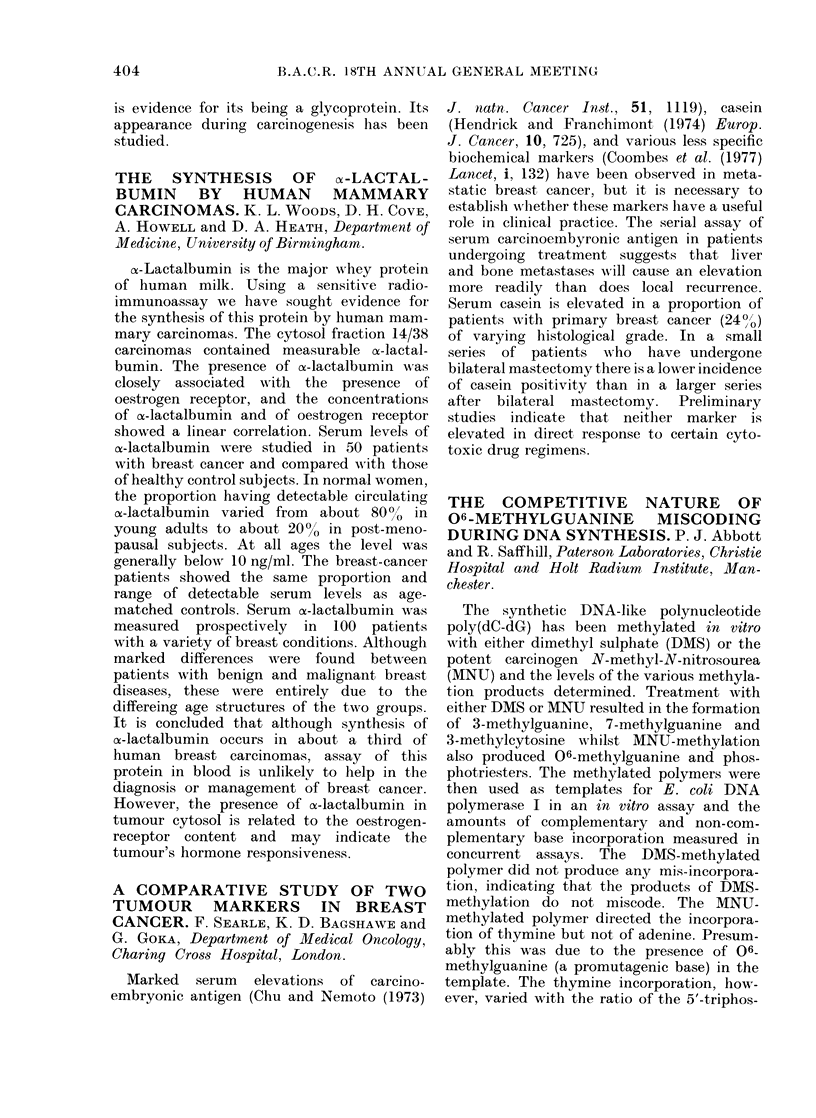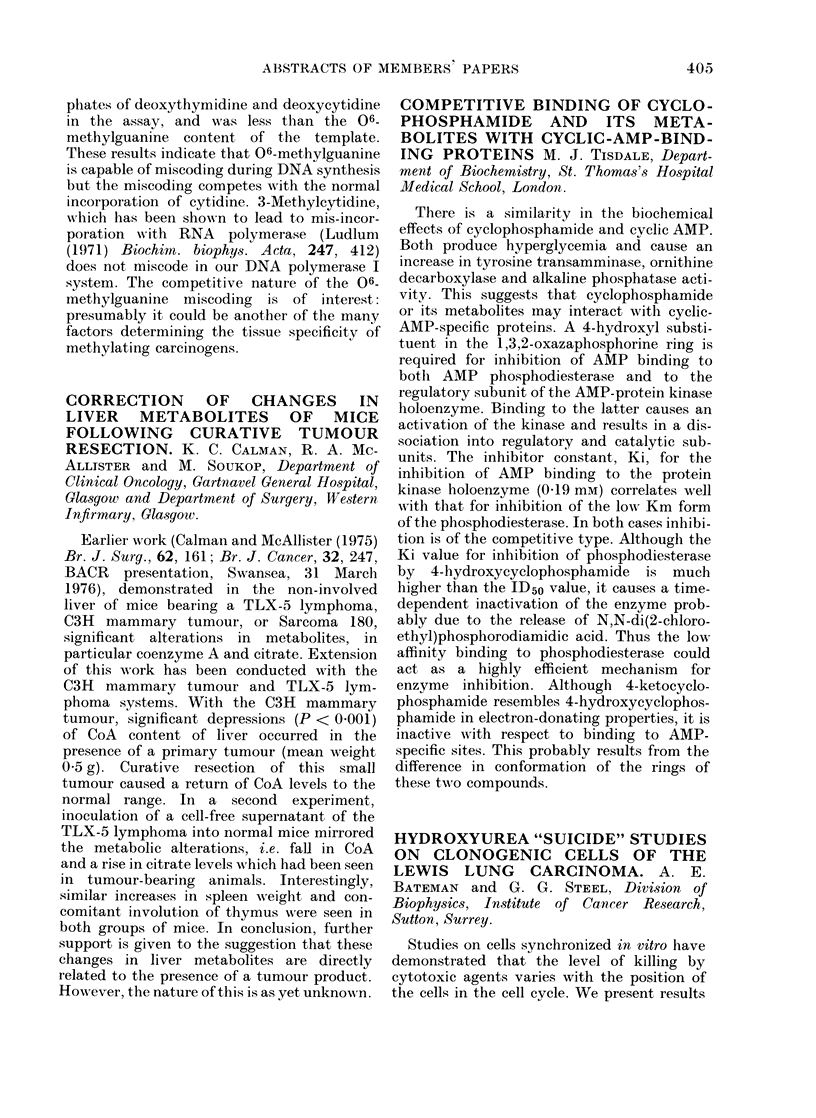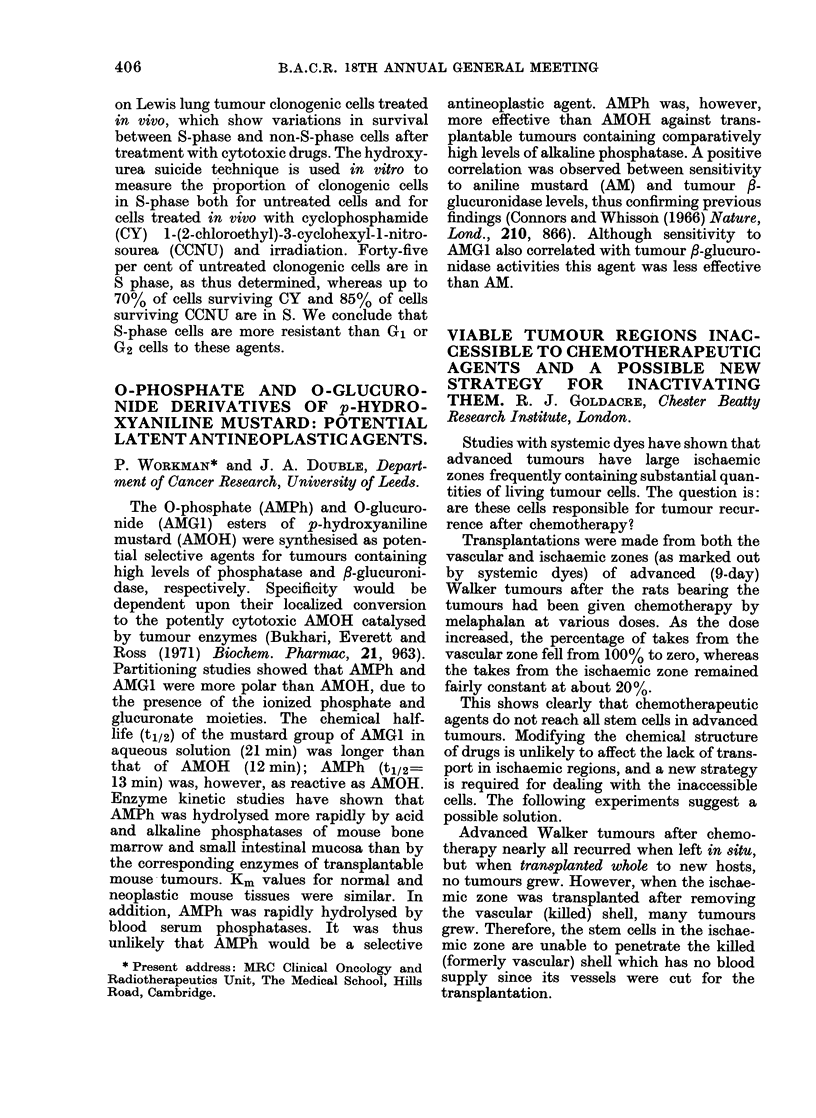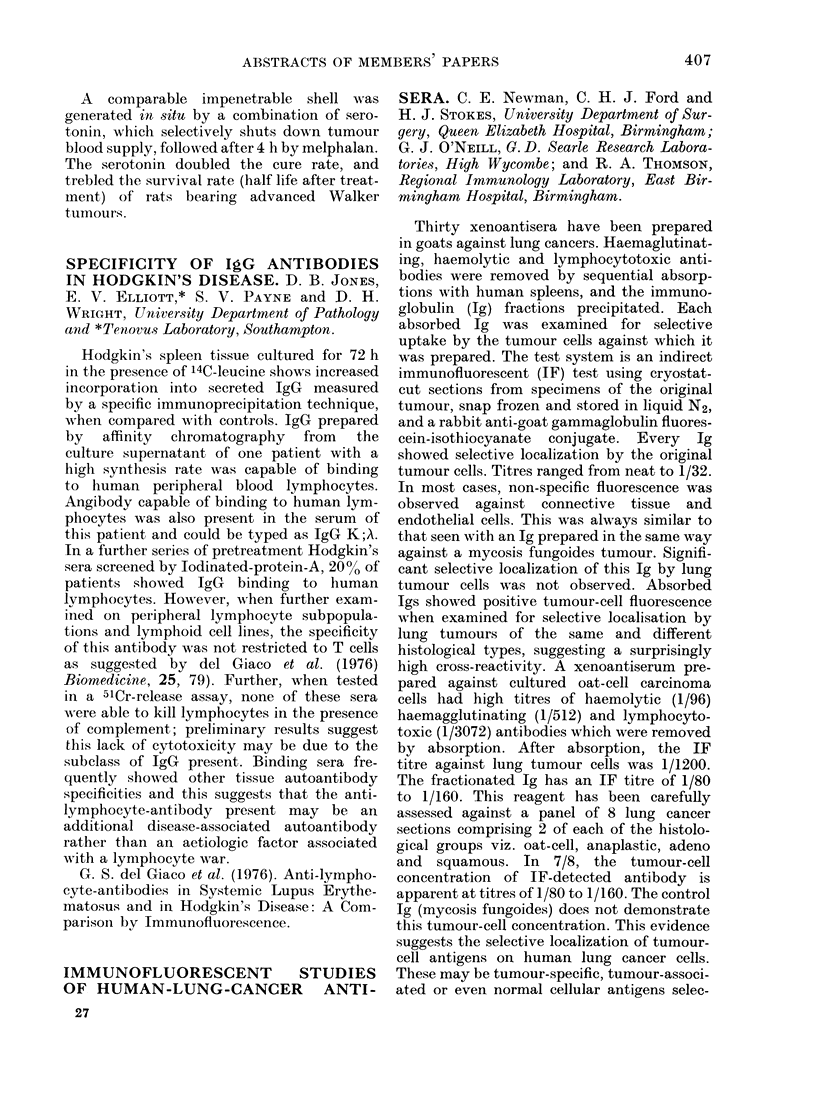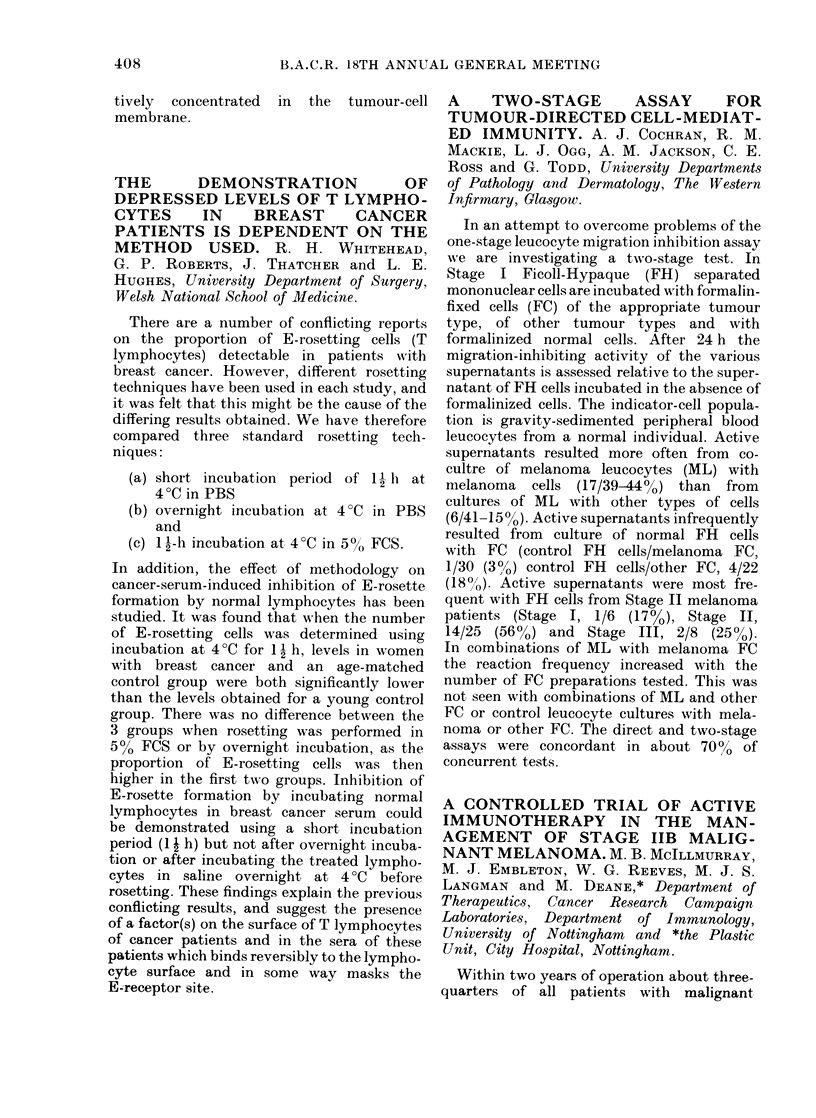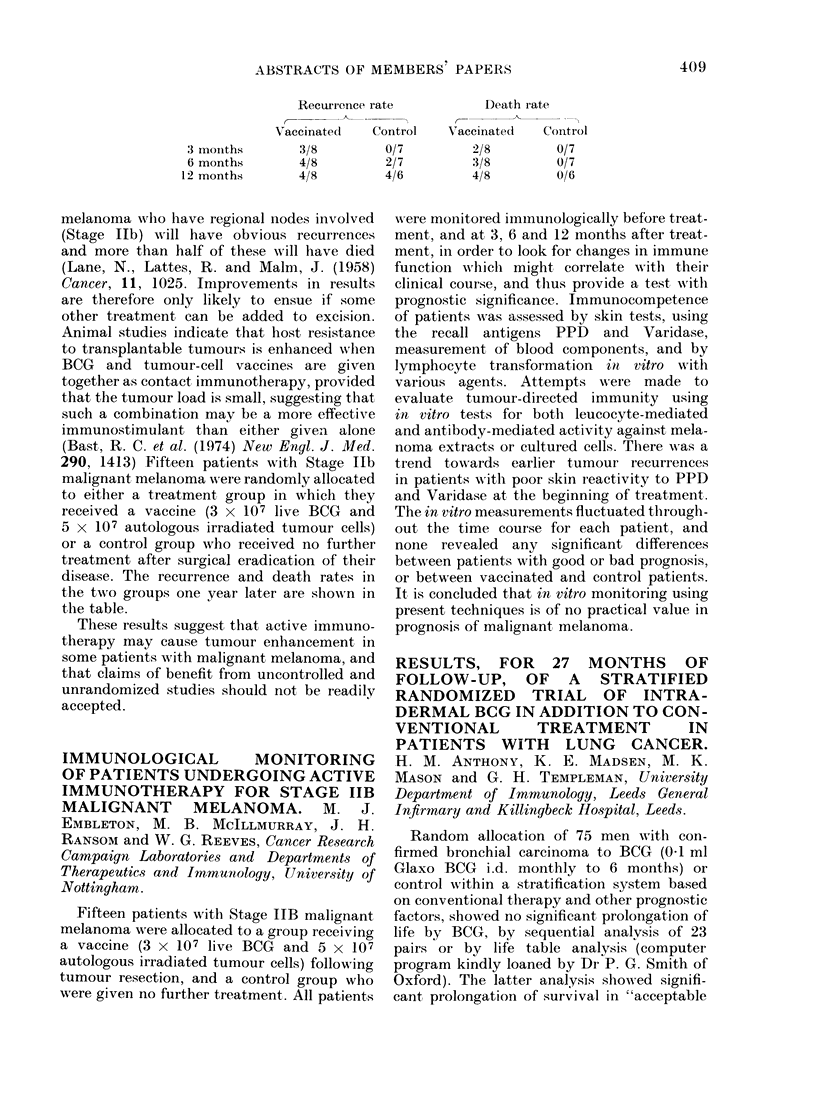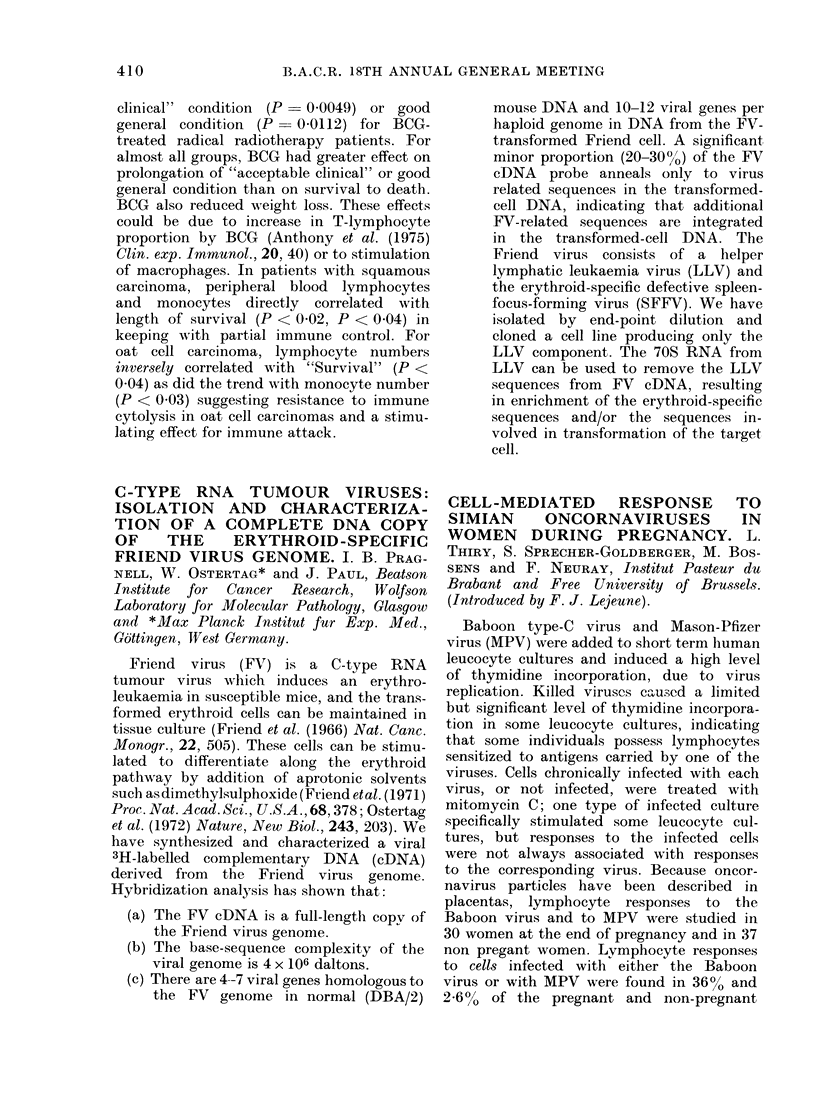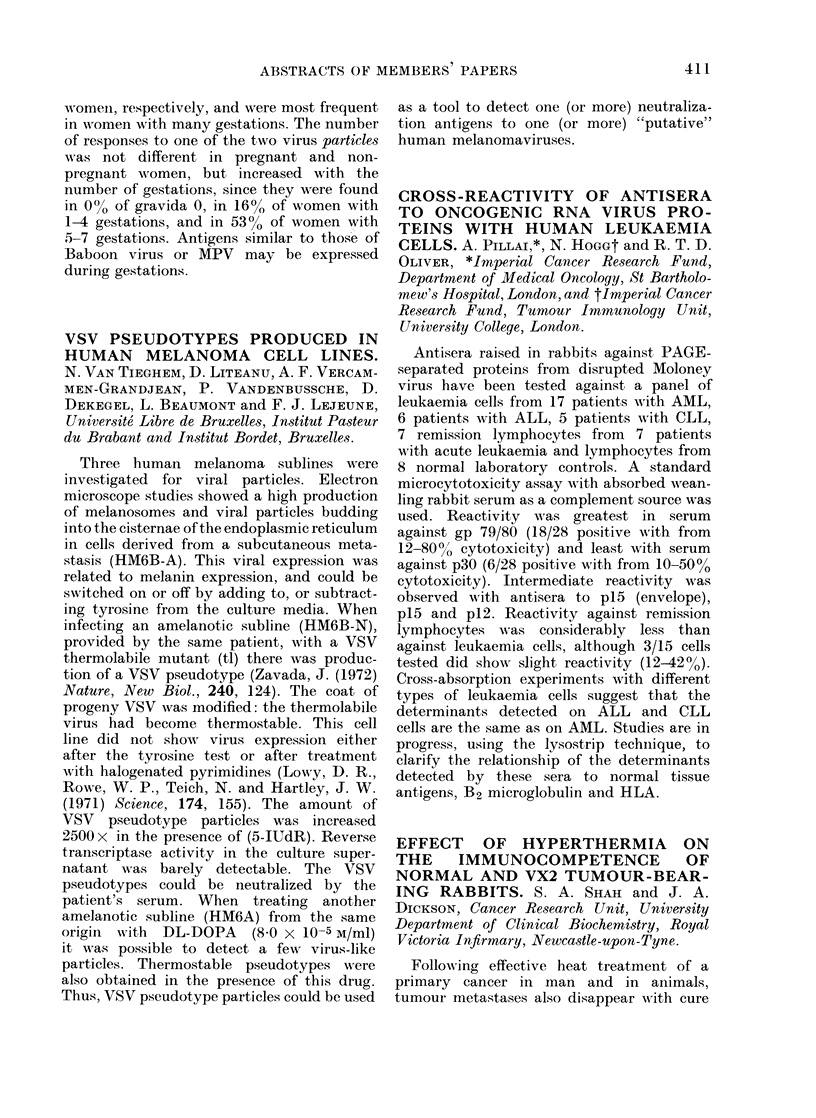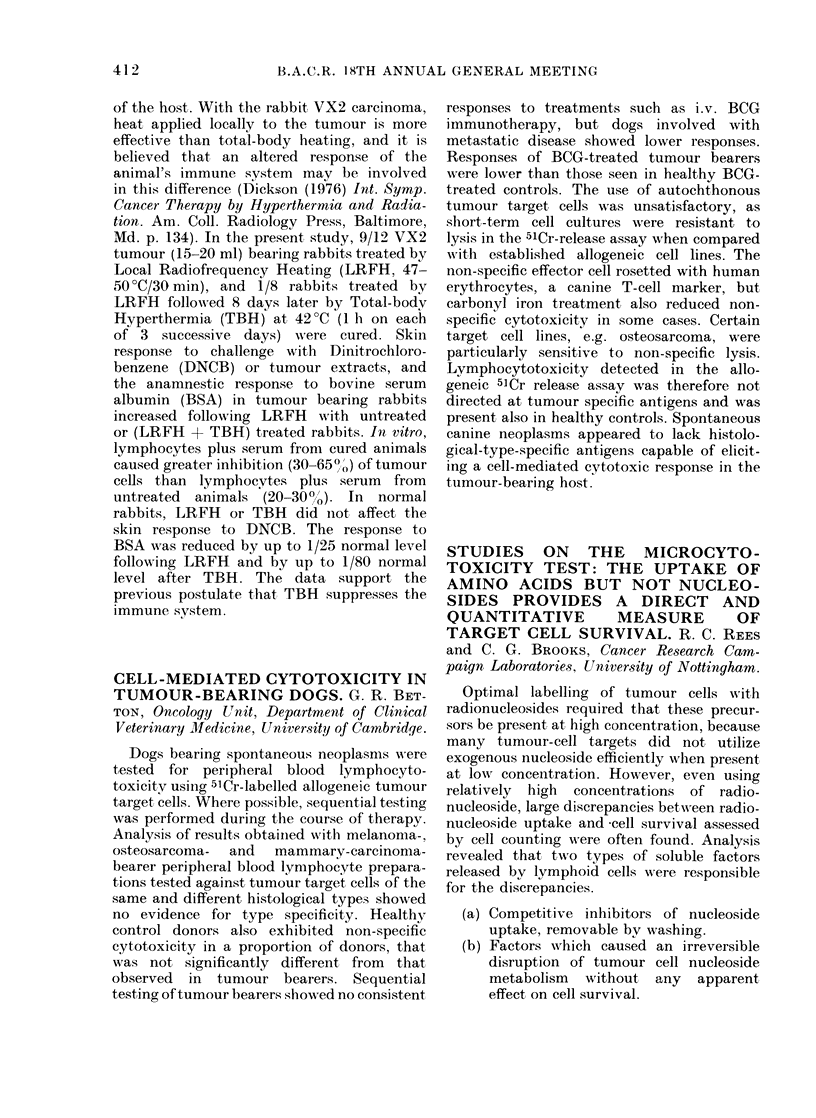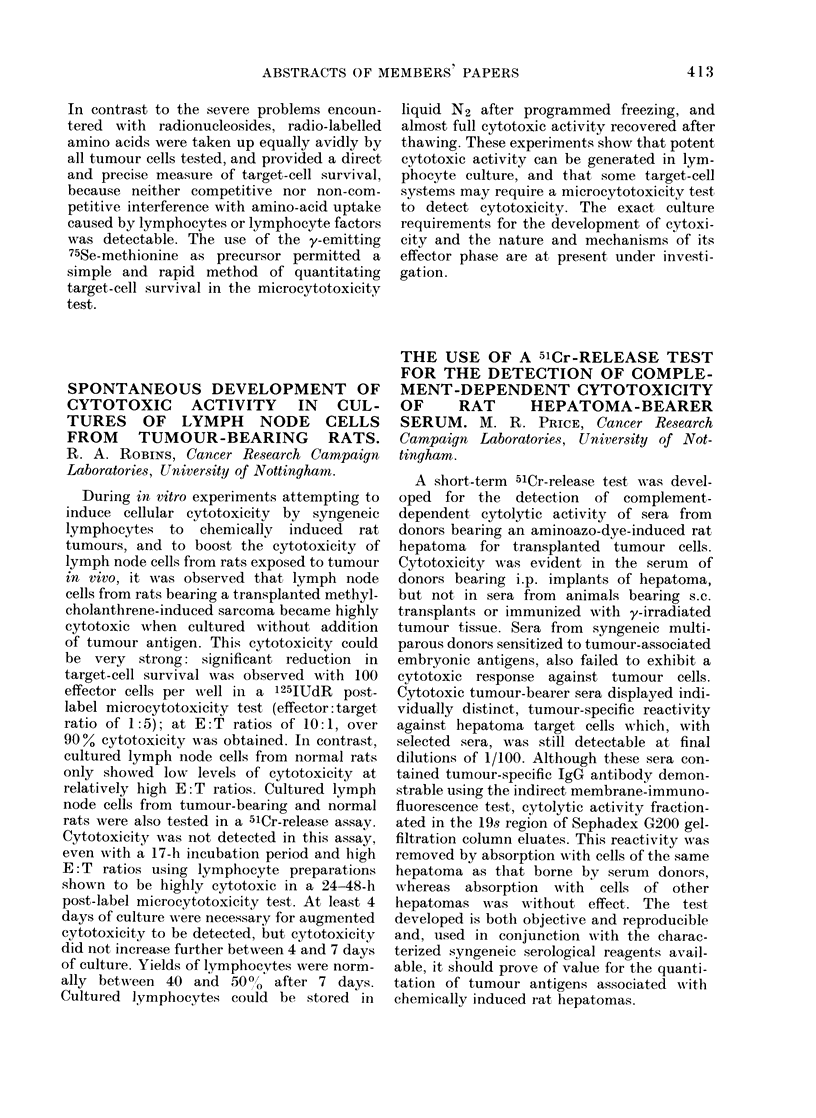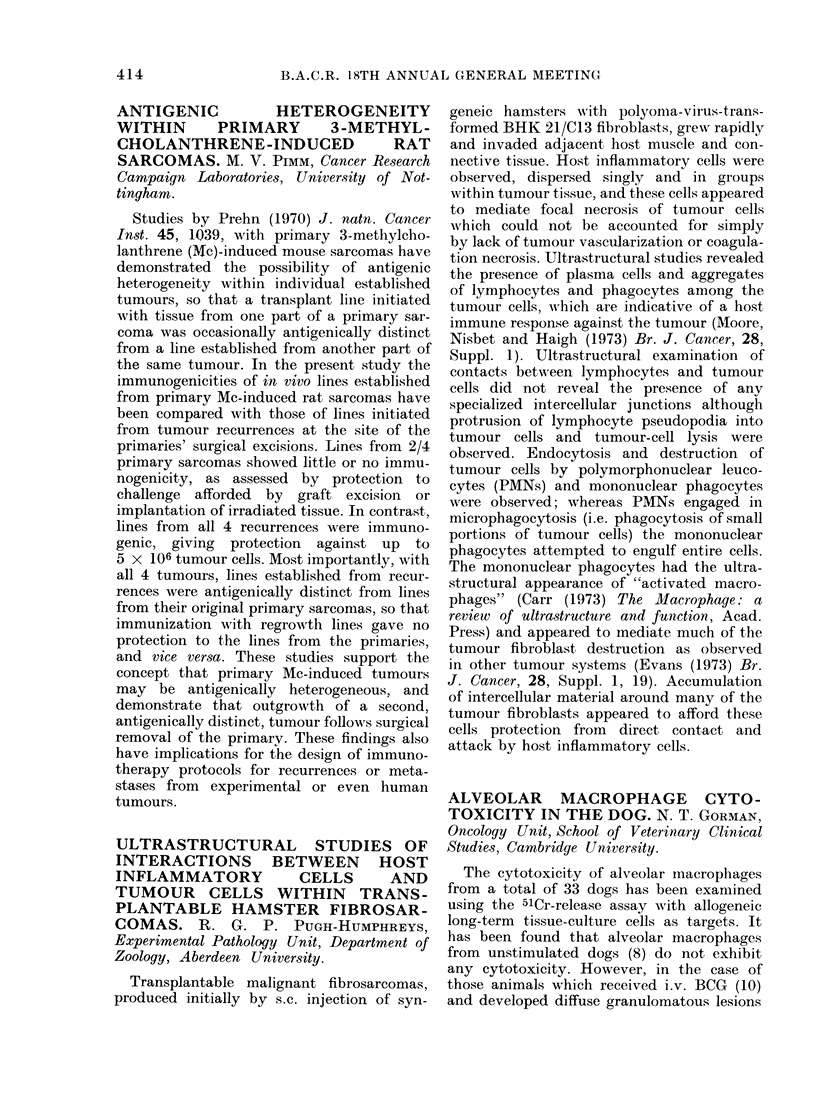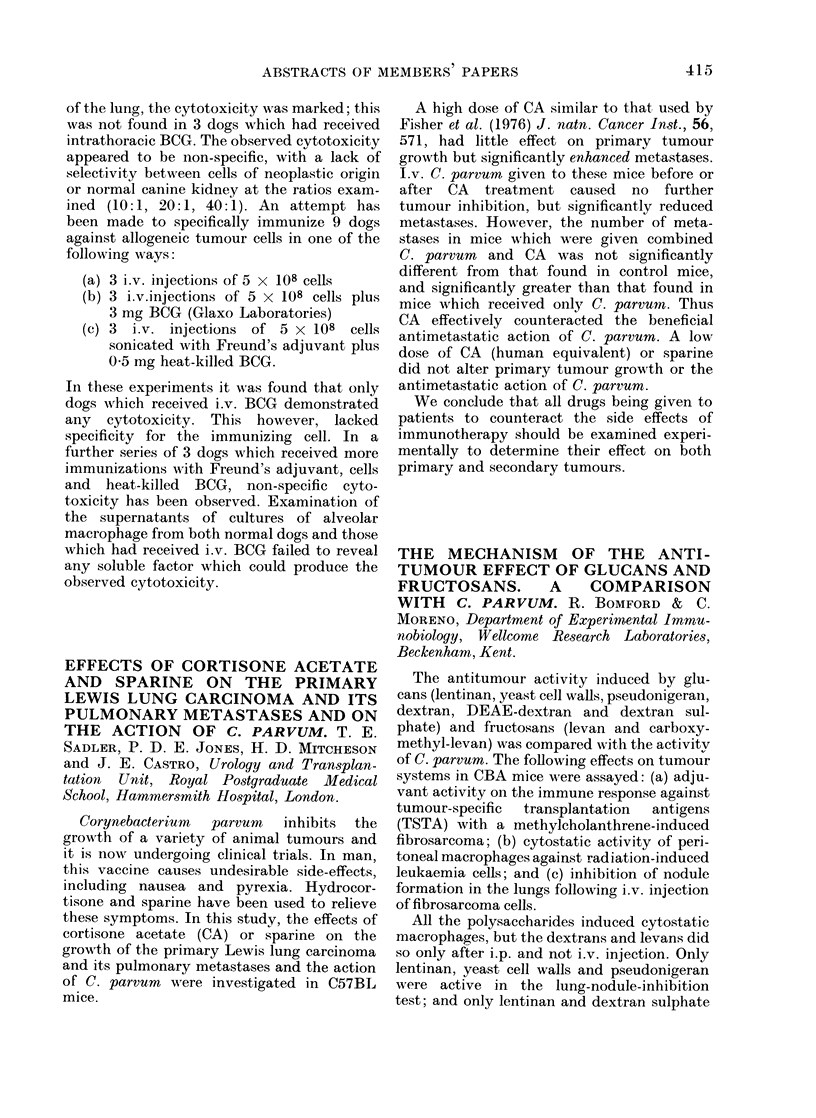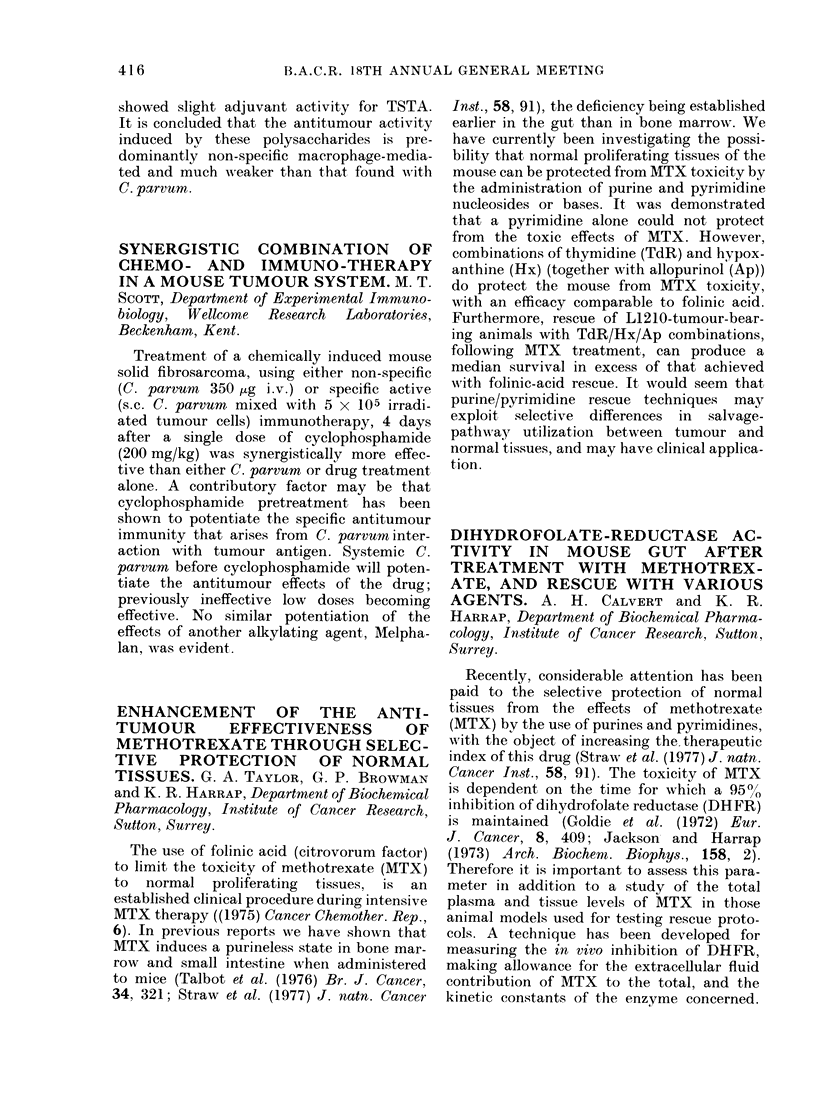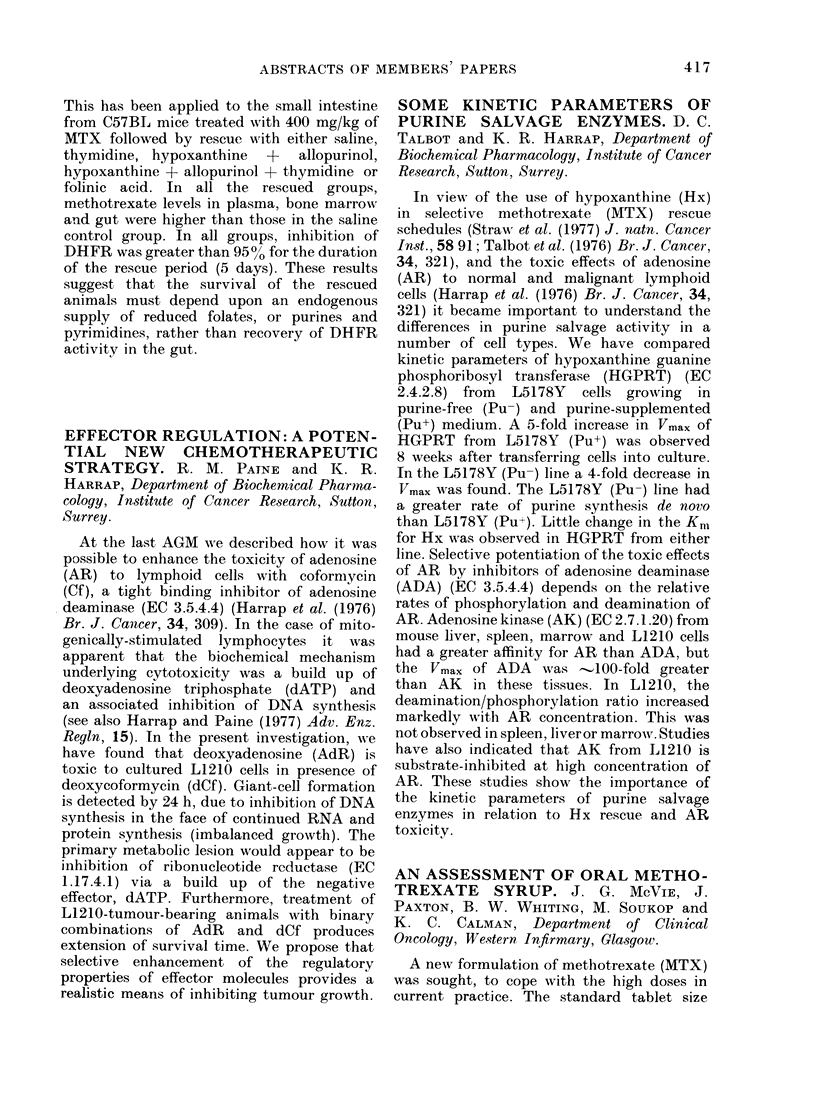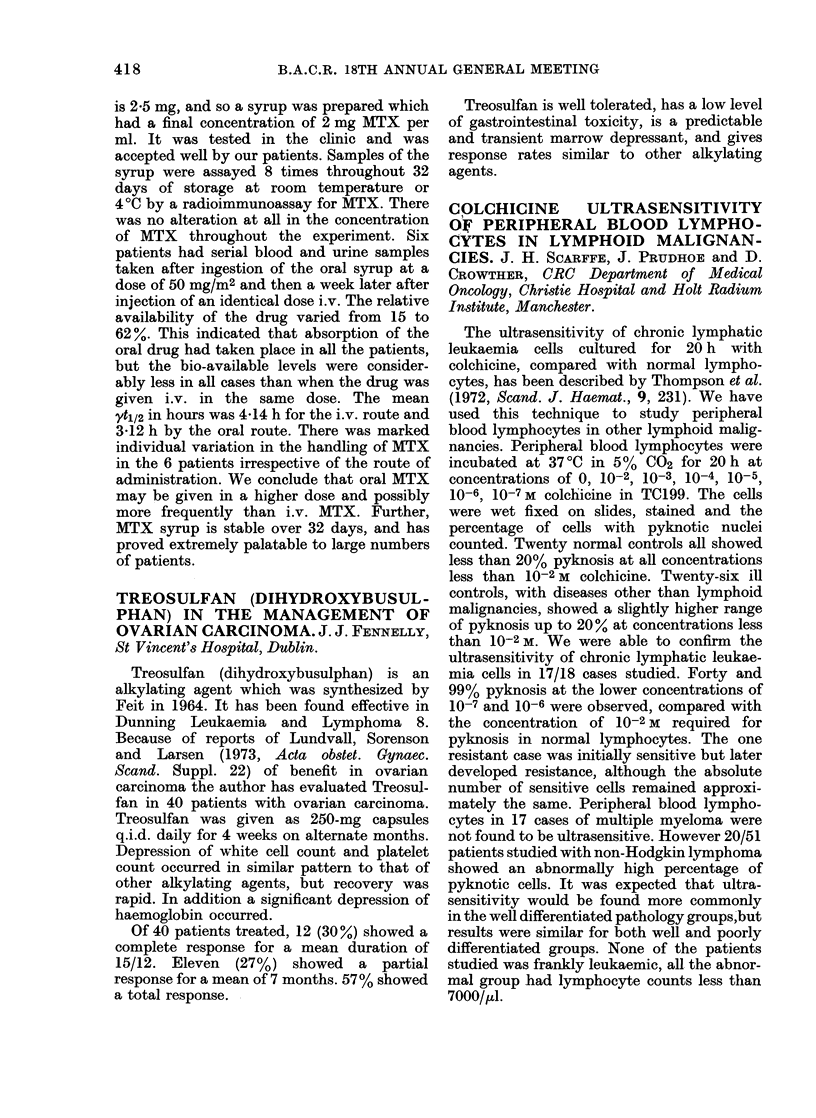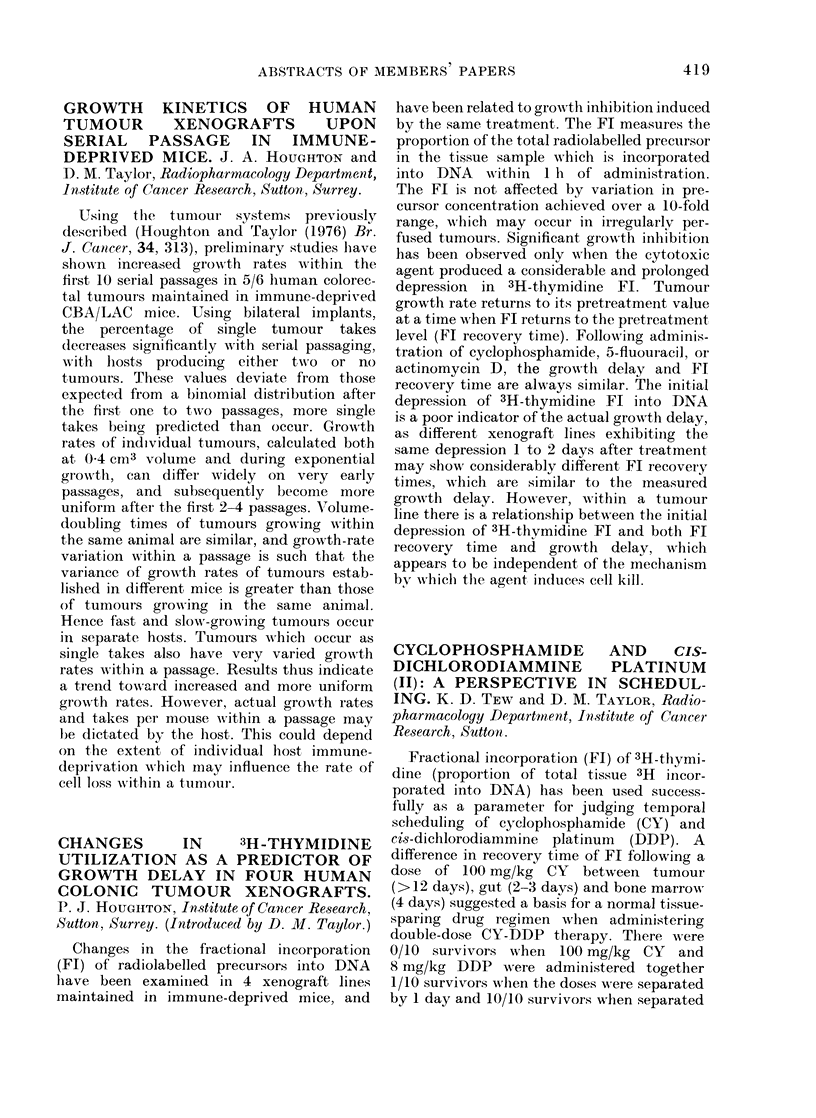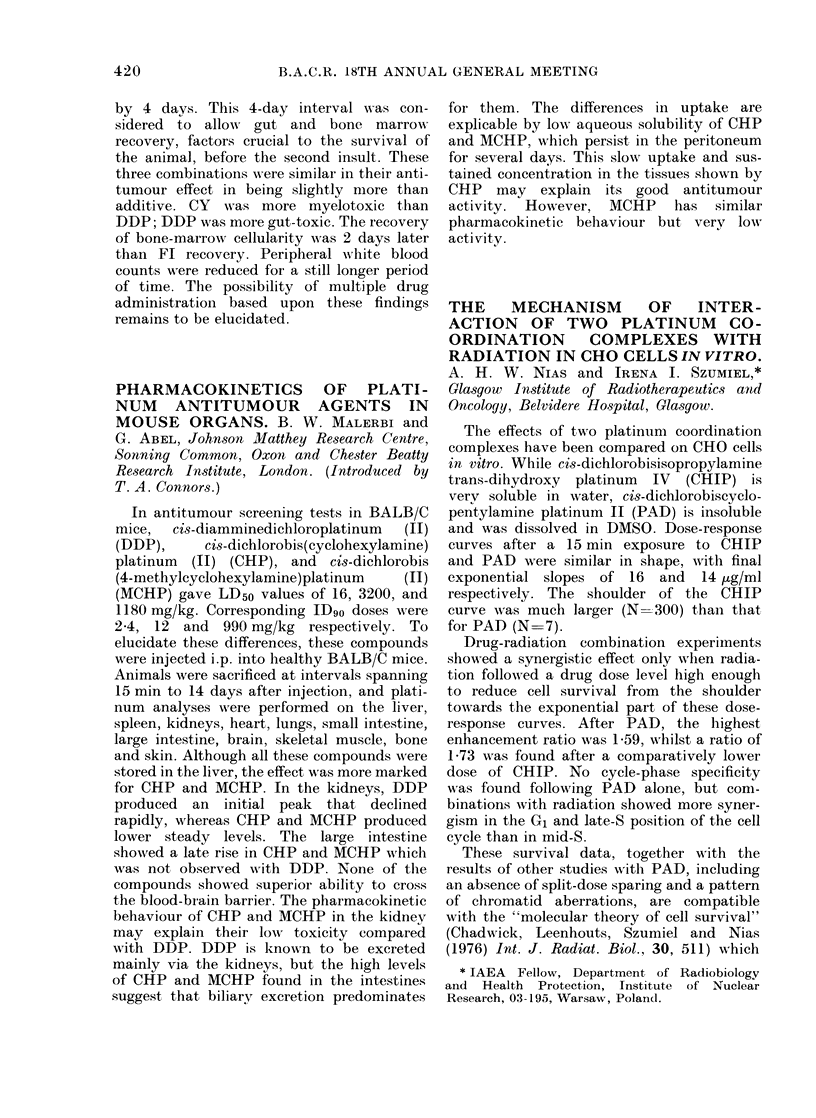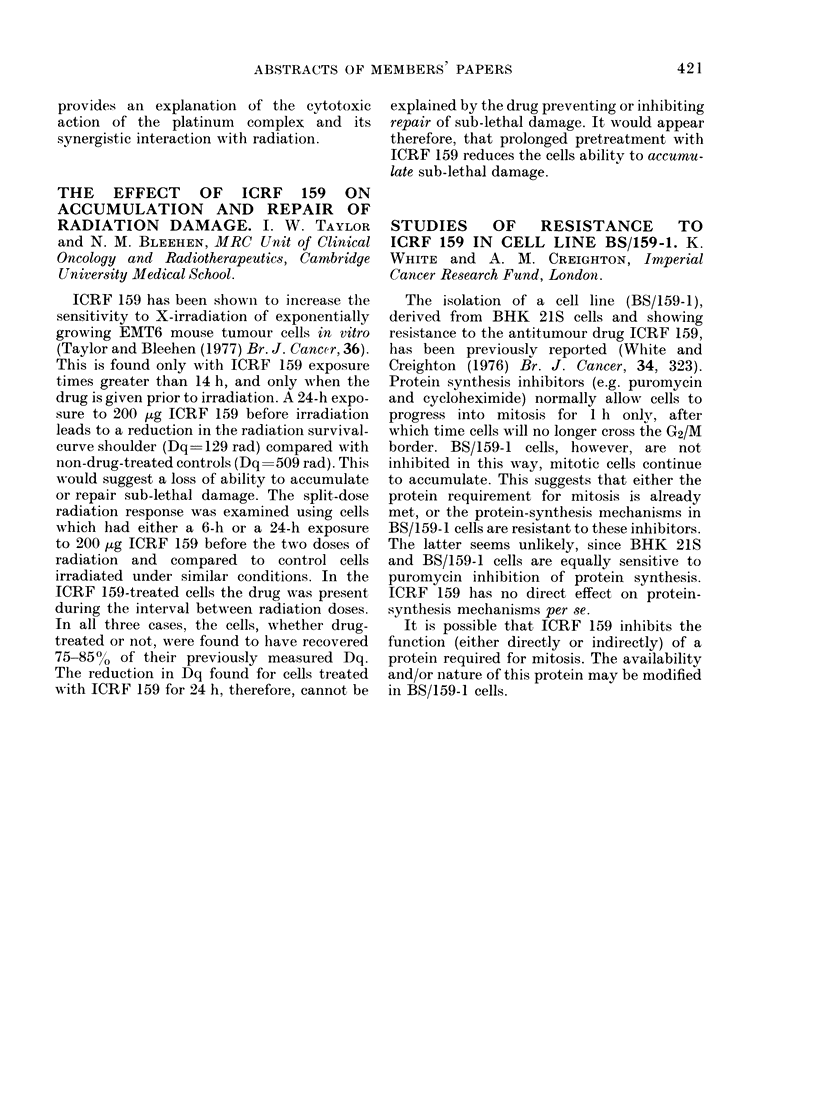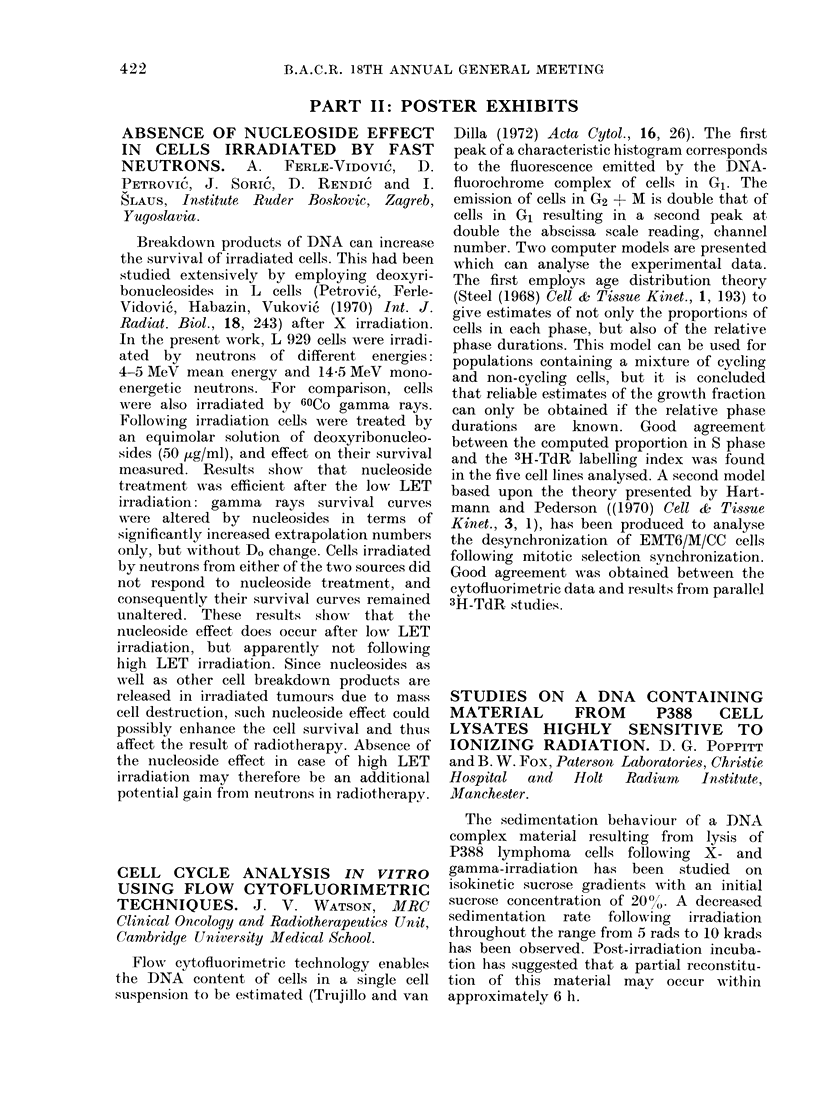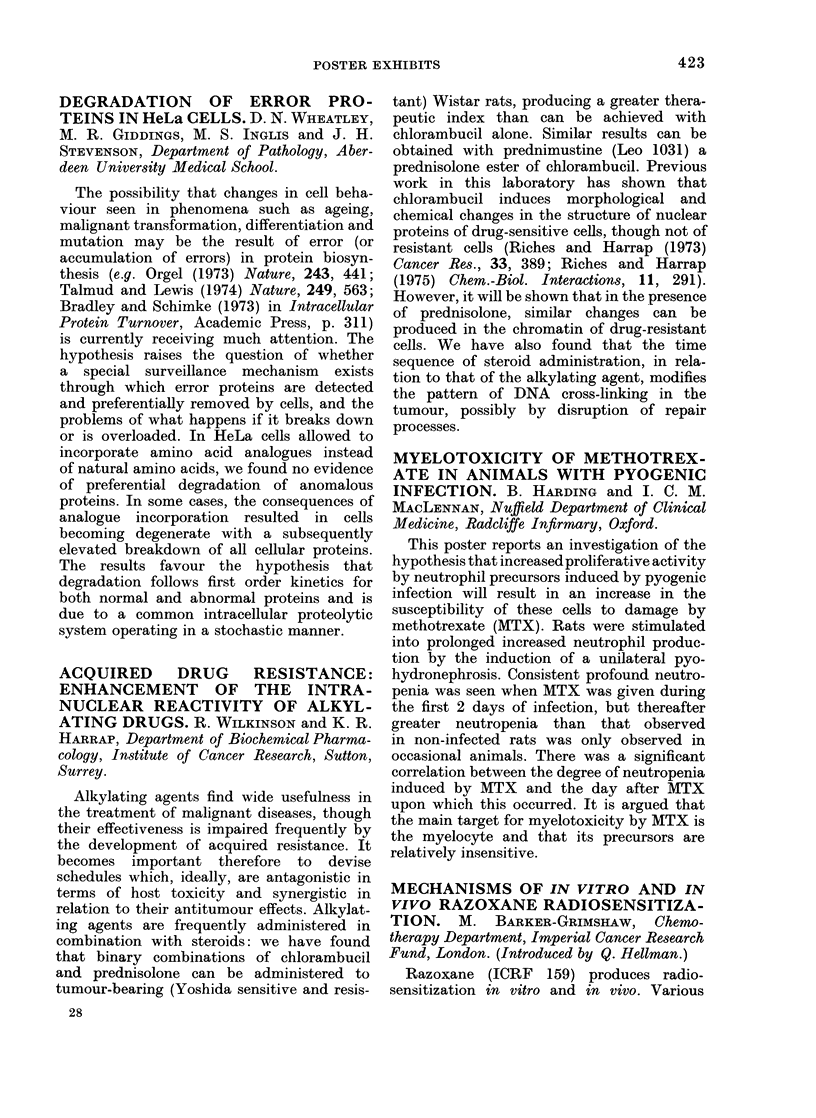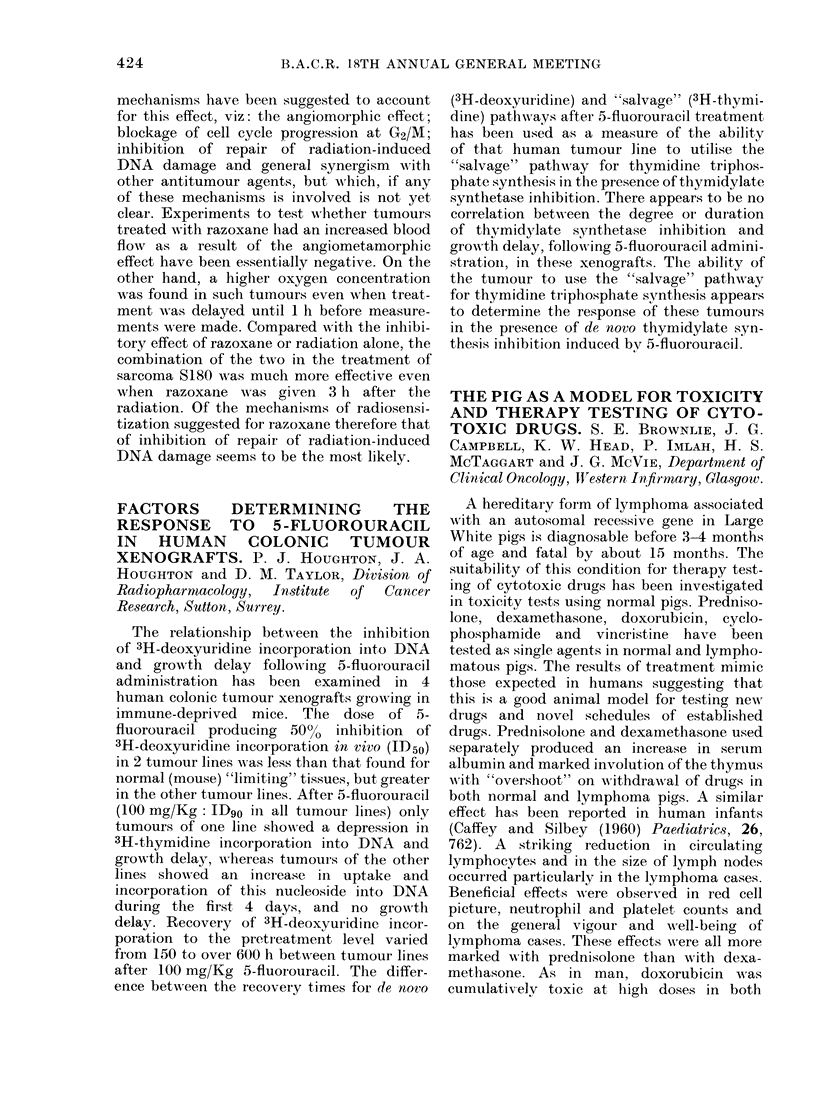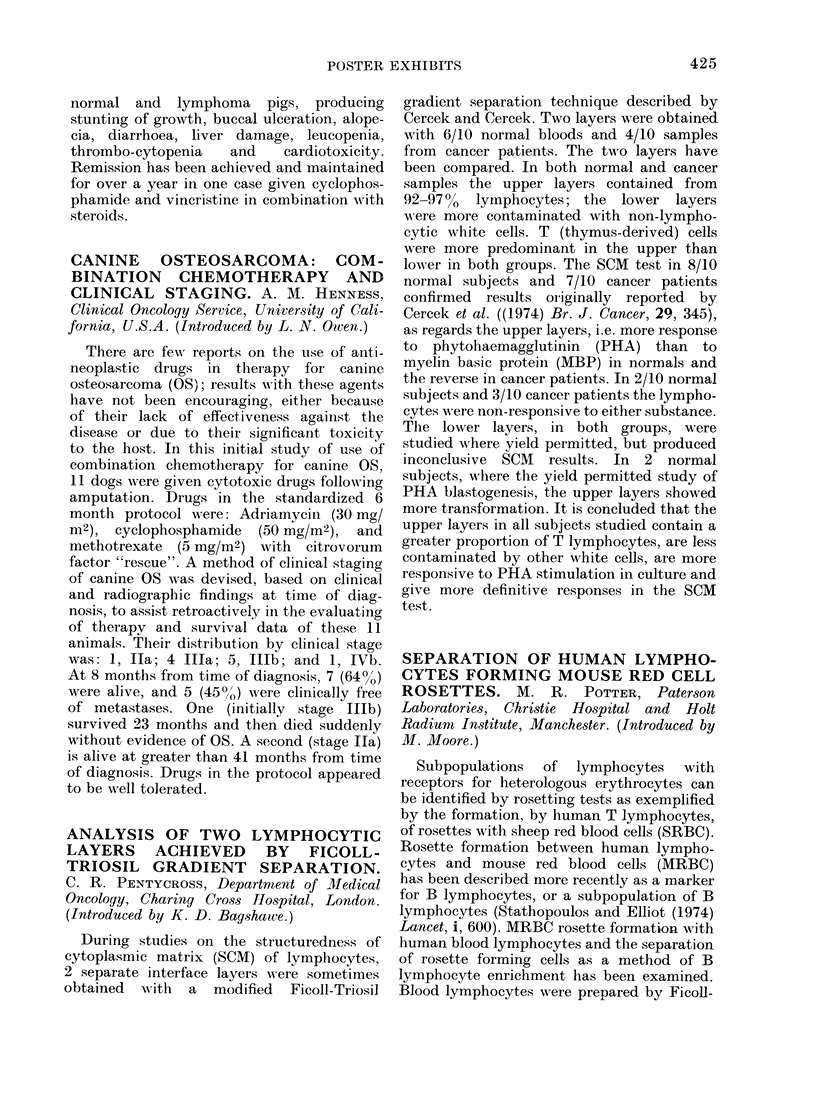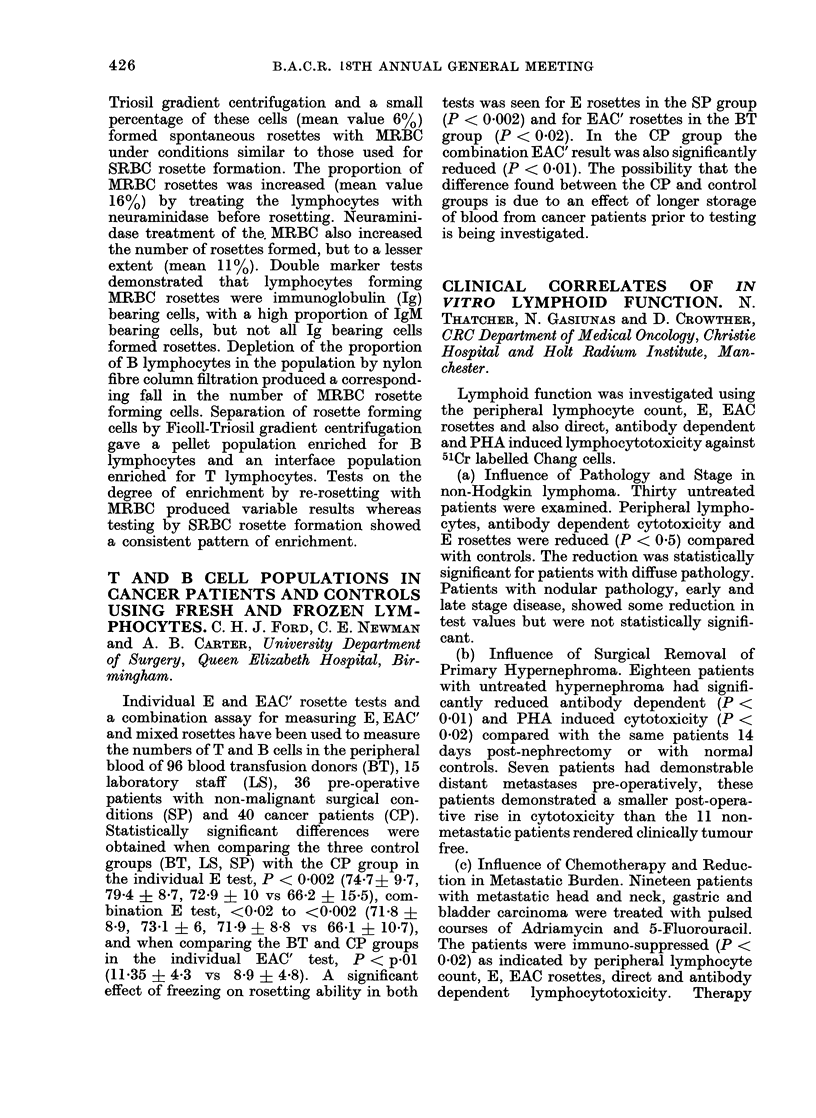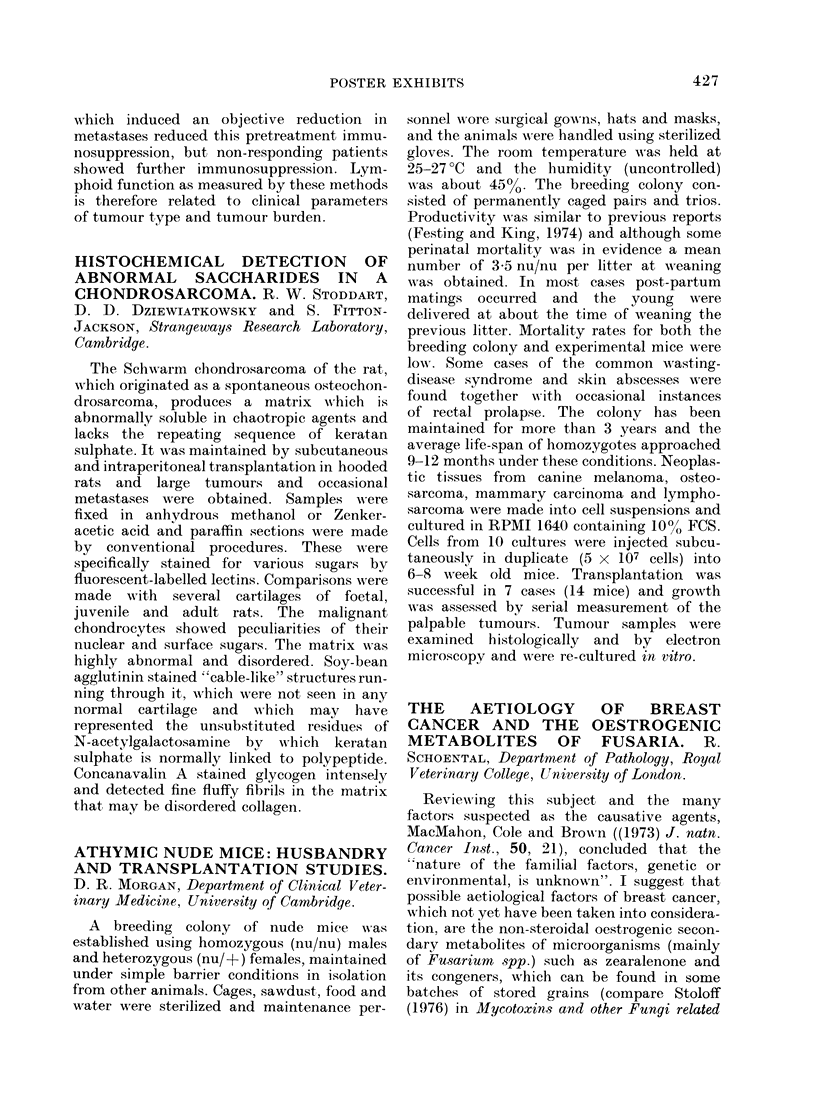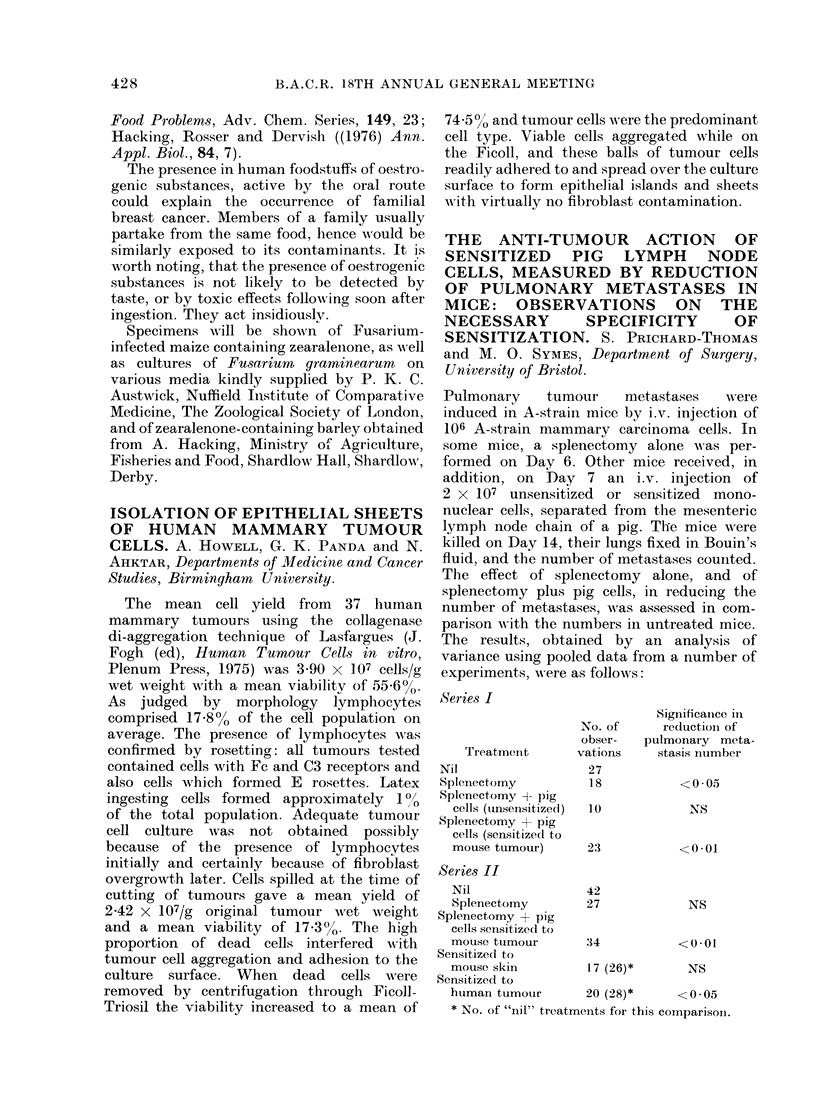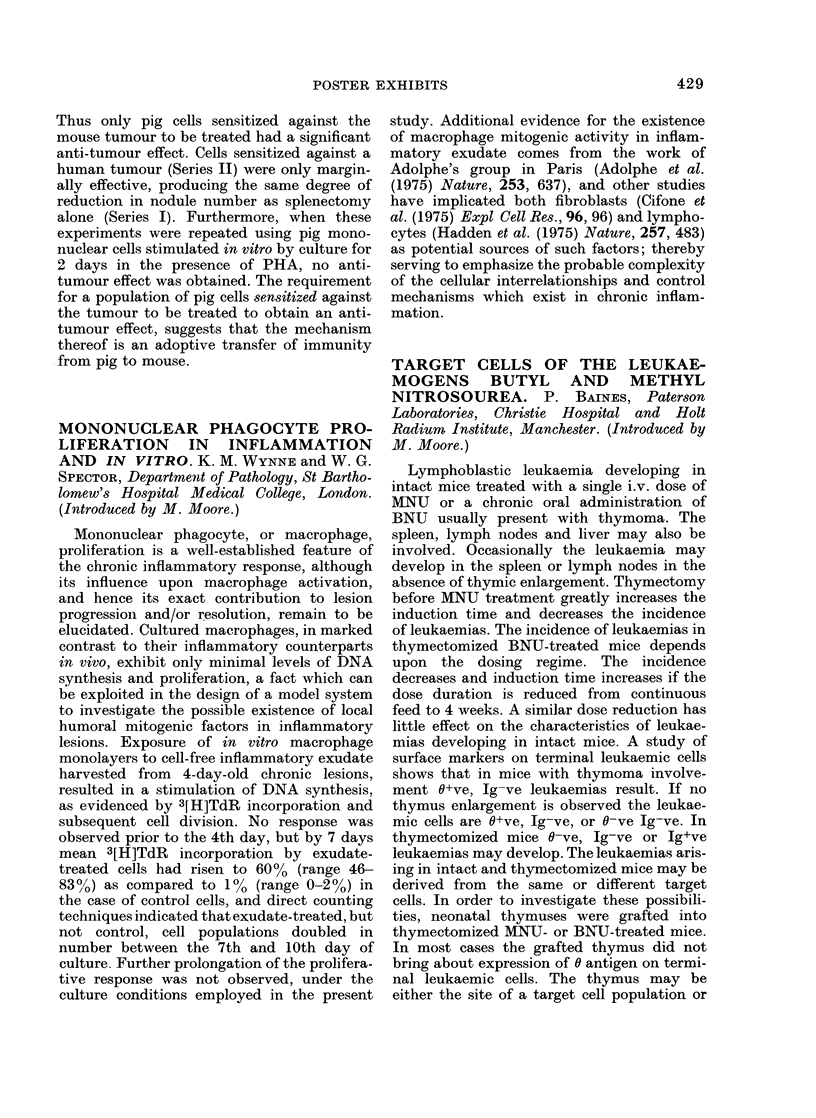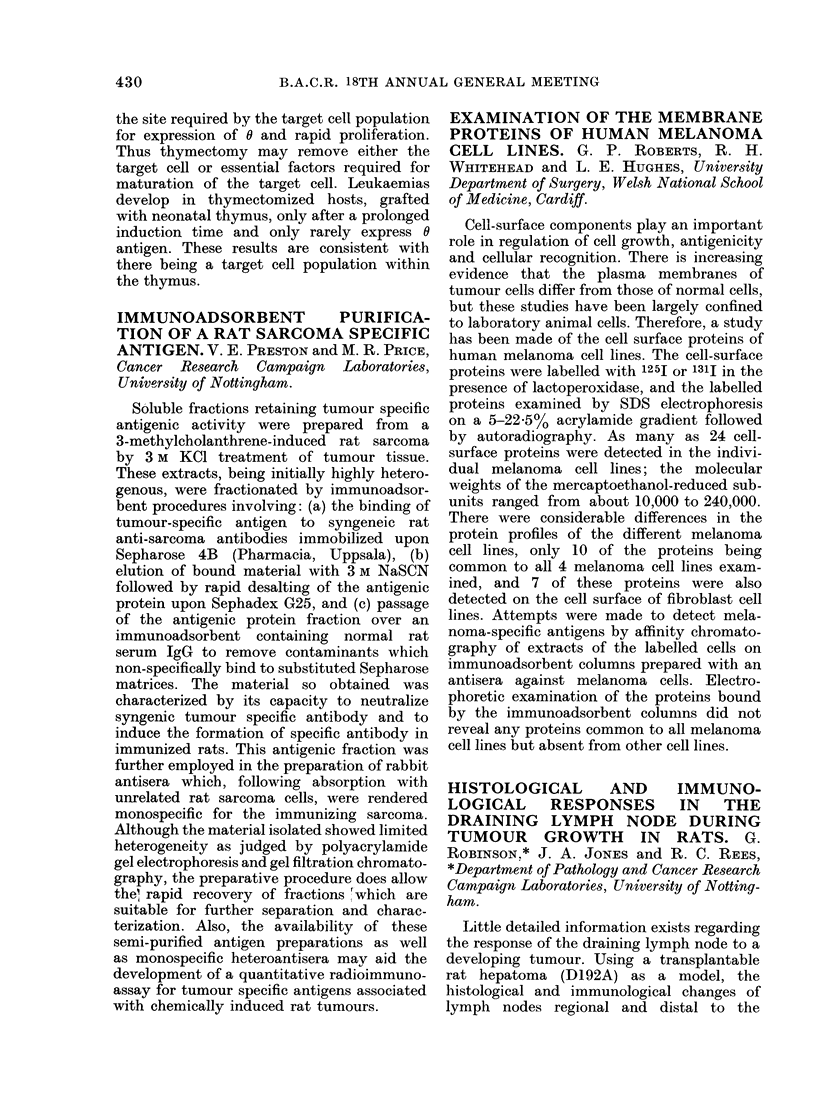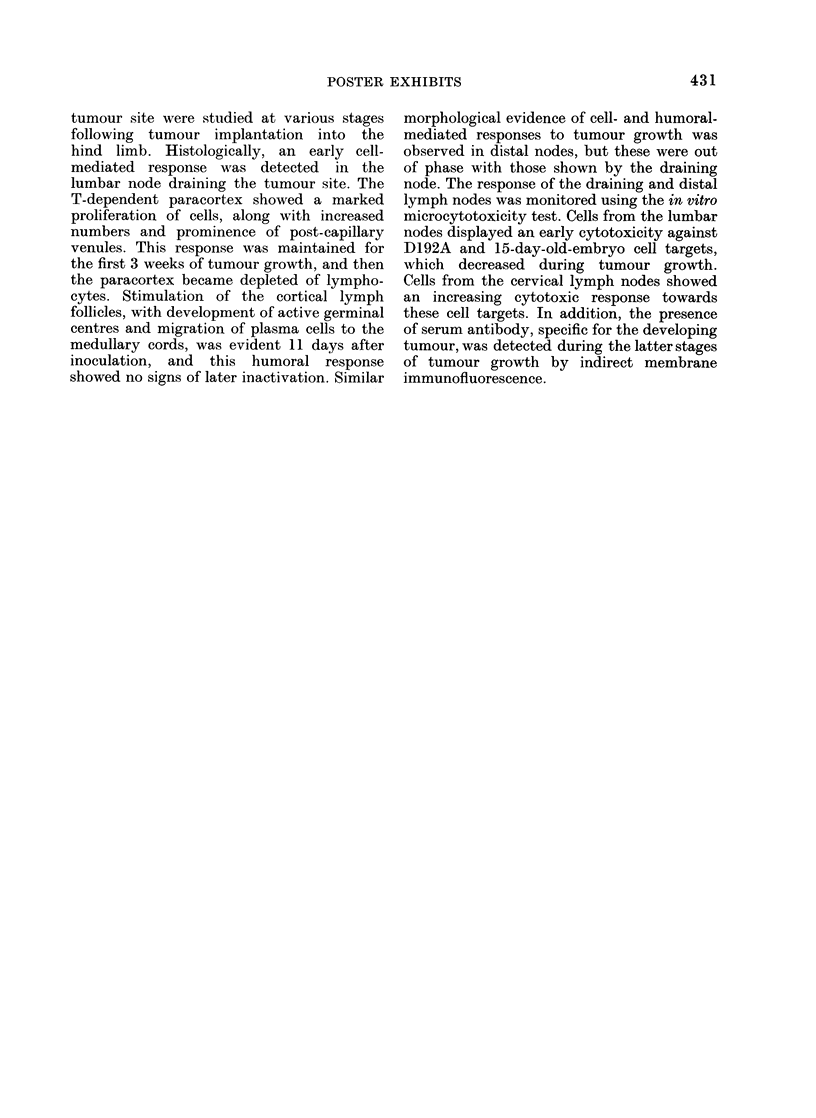# B.A.C.R. 18th Annual General Meeting

**Published:** 1977-09

**Authors:** 


### PART I: ABSTRACTS OF MEMBERS' PROFFERED PAPERS

### ENHANCEMENT OF CHEMICALLY-INDUCED NEOPLASIA BY PROXIMAL ENTERECTOMY.

R. C. N. WILLIAMSON, F. L. R. BAUER and R. A. MALT, *United Bristol Hospitals and Massachusetts General Hospital, Boston, U.S.A.* (*Introduced by M. O. Symes*).

Azoxymethane-induced tumours in the rat, morphologically indistinguishable from those arising in man, affect the duodenum, jejunum and colon, but usually spare the ileum (Ward, J. M. (1975) *Vet. Pathol.*, **12**, 165). Proximal small-bowel resection (PSBR) causes prompt ileal hyperplasia, with a lesser response in the colon. To test the possible adjuvant effect of postresectional hyperplasia on intestinal carcinogenesis, rats ($n = 76$) were submitted to 50% PSBR 10 days after the last of 16 weekly injections of azoxymethane (10 mg/kg s.c.) or vehicle. Controls were unoperated. Nucleic acid contents of upper ileal mucosa in rats receiving vehicle alone showed increments of 76% (RNA) and 68% (DNA) 3 months after PSBR ($P < 0{\cdot}001$). The number and distribution of intestinal tumours were noted in rats sacrificed at 30 weeks, or dying of cancer in the preceding 4 weeks. Intestinal tumours occurred in all but 1 of the rats receiving azoxymethane, but in none of those injected with vehicle. Proximal enterectomy raised the incidence of colonic tumours but failed to induce ileal carcinogenesis, despite promoting marked mucosal hyperplasia. We conclude that postresectional hyperplasia cannot overcome the relative insusceptibility of the ileum to neoplasia, but that it enhances the induction of tumours in colon previously exposed to chemical carcinogens.

No. tumours per rat (after azoxymethane)

| | | Duodenojejunum | Ileum | Colon | Ear canal |
|---|---|---|---|---|---|
| Control | 18 | 1·3 | 0·1 | 1·6 | 0·8 |
| PSBR | 16 | 0·8 | nil | 2·9 | 1·2 |
| | | NS | NS | $P<0{\cdot}02$ | NS |

### AN INVESTIGATION OF ETHYLNITROSOUREA-INDUCED CARCINOGENESIS IN THE RAT BRAIN BY AN *IN VIVO–IN VITRO* METHOD.

J. P. ROSCOE and P. J. CLAISSE, *Department of Cell Pathology, School of Pathology, Middlesex Hospital Medical School.*

Pregnant rats were injected with ethylnitrosourea to induce cerebral gliomas in a high proportion of the offspring. With a dose of 40–50 mg/kg body weight the average latent period for these brain tumours is 246 days. Cultures have been prepared at different times after transplacental exposure but before a tumour is visible. It has been reported previously (Roscoe and Claisse (1976) *Nature, Lond.*, **262**, 314) that there is a marked difference in the behaviour of cultures prepared 138–145 days p.i. and 2 days p.i. These experiments have been extended and are reported here. Cultures prepared 111–112 days p.i. were similar to those derived 138–145 days p.i. They contained cells like those found in tumour cultures which can predominate in culture and are tumourigenic. Similar cells observed in cultures prepared 90–91 days p.i. appear to be lost on sub-culturing. These cells were not seen when cultures were initiated at still earlier times (60, 34–35, 5, 3, 2 days p.i.). However it has been shown that cultures prepared 2 days p.i. yield cells which are tumourigenic on prolonged culturing. These results provide a framework for further analysis of the latent period and more detailed studies are in progress.

### FIBRINOLYTIC ACTIVITY ASSOCIATED WITH RAT BRAIN CELLS EXPOSED TRANSPLACENTALLY TO THE CARCINOGEN ETHYLNITROSOUREA.

T. A. HINCE and J. P. ROSCOE, *Department of Cell Pathology, School of Pathology, Middlesex Hospital Medical School.*

An increased fibrinolytic activity of tumour and transformed cells, as a result of increased amounts of plasminogen activator, has been proposed as another marker for transformed cells (Jones *et al.* (1976) *Cancer Res.*, **36**, 2863). In this study we have investigated the fibrinolytic activity of cell lines derived from cerebral gliomas of the rat brain and cultures derived from the brains of rats after their transplacental exposure to the carcinogen ethylnitrosourea (ENU) using an *in vivo/in vitro* system (Roscoe and Claisse, 1976, *Nature, Lond.*, **262**, 314). Using a fibrin-overlay method (Jones *et al.*, 1975, *Cell*, **5**, 323) we have demonstrated that cell lines derived from a cerebral glioma and from rat brains 111–112 days after their exposure to the carcinogen show a very high level of fibrinolytic activity. In contrast, control cells and cultures derived from the brains of animals exposed to buffer alone show low levels of activity. A detailed study of the relationship between fibrinolytic activity, measured as the percentage of total colonies giving lysis, and cell colony size indicated that there was a marked difference between transformed and control cells. Transformed cells showed a rapid rise in fibrinolytic activity with increasing colony size and reached a plateau level $>70\%$ at colony sizes of below 70 cells/colony; whereas control cells showed a linear increase with colony size and much reduced levels of activity: $<30\%$ at larger colony sizes, $>130$ cells-colony. Thus in this system an increased amount of fibrinolytic activity is associated with malignant transformation.

### SV40-3T3 CELL PLASMINOGEN ACTIVATOR-MEDIATED INITIATION OF MITOSIS IN QUIESCENT 3T3 CELLS.

P. WHUR, M. GORDON, D. C. WILLIAMS, C. URQUHART and E. WRIGHT, *Marie Curie Foundation, Oxted, Surrey and Imperial College, London and Royal Free Hospital, Medical School, London.*

The number of dividing cells in a population of quiescent 3T3 cells in low serum increases significantly in the presence of SV40-3T3 cells and added plasminogen. This effect is attributable to plasminogen activation (Whur *et al.* (1976) *Nature, Lond.*, **260**, 709). The effect of plasminogen activation on mitosis decreases as serum stimulation becomes optimal, suggesting that the former may potentiate the latter. Scanning electron micrographs show that plasminogen activation causes fissures to open between previously confluent 3T3 cells; thus the diffusion boundary layer may become disrupted, leading to a reinitiation of mitosis by serum growth factors. This mechanism may also operate *in vivo*. Tumour cells would initiate a local inflammatory response with consequent leakage of serum; the plasminogen would then be locally activated by the tumour cells which would then divide at a faster rate.

### CONTROL OF HAEMOPOIETIC STEM-CELL POPULATIONS.

E. G. WRIGHT, *Paterson Laboratories, Christie Hospital and Holt Radium Institute, Manchester.* (*Introduced by R. Schofield*).

Haemopoietic cells are derived from common pluripotential spleen-colony-forming stem cells (CFU-S). The majority of these stem cells, in the normal steady state, are not proliferating, but can be stimulated into cell cycle by a decrease in their population size. The nature of CFU-S proliferation control is not known. There is, however, evidence that it is "local" rather than humoral. The presence of proliferating and non-proliferating stem cells in, respectively, the bone marrow and spleens of phenylhydrazine (PHZ)-treated mice (Rencricca *et al.* (1970) *Blood*, **36**, 764) has afforded the opportunity to investigate the presence and role of local factors responsible for the control of CFU-S proliferation. This has been done by measuring the proliferative activity of femoral and splenic CFU-S resulting from the addition of radiation-killed splenic or bone-marrow cell populations. When bone-marrow cells from PHZ-treated mice are incubated with irradiated spleen cells taken from the same mice, there is a marked fall in the proportion of femoral CFU-S in DNA synthesis. In the converse experiments, rapid triggering of splenic CFU-S is achieved. Changes in CFU-S proliferation have also been demonstrated in other situations, where cell populations containing proliferating and non-proliferating CFU-S are mixed. It is not, therefore, a phenomenon specifically related to the PHZ-treated mouse. The effects on the proliferative activity of CFU-S resulting from the incubation of haemopoietic cells with irradiated cell populations suggest that some part or parts of these populations contain material capable of altering the rate of stem-cell proliferation. It seems probable that these findings represent some aspect of the local physiological CFU-S proliferation-control process.

**THE SPECIFIC STAINING OF SUGARS IN THE HISTOCHEMICAL ANALYSIS OF BONE MARROW AND THE MYELOID LEUKAEMIAS.** R. W. STODDART, W. JACOBSON and R. D. COLLINS, *Strangeways Research Laboratory, Cambridge, and Department of Pathology, Vanderbilt University, Nashville, Tennessee, U.S.A.*

In many glycoproteins of cellular surfaces, mannosyl residues lie in the "cores" of the oligosaccharides, while sialic acid is always at the non-reducing (exterior) terminals. Galactosyl groups are usually either terminal or sub-terminal to sialic acid. Fluorescent-labelled concanavalin A (FL-ConA) can be used to stain for $\alpha$-mannosyl (or $\alpha$-glucosyl) groups; the similar derivatives of *Ricinus communis* haemagglutin (FL-RCA) and of aprotinin (FLA) stain for $\beta$-galactosyl groups and sialyl groups respectively. Fresh smears, or paraffin sections of methanol-fixed bone marrow were used. Myeloblasts, myelocytes and leucocytes showed staining of their surfaces, cytoplasm and nuclear membranes with all three stains. Granules in eosinophils and basophils stained in each case, but those of neutrophils bound only FL-ConA. In megakaryocytes, there was intense cytoplasmic staining with FL-RCA and FL-ConA; the platelets stained strongly with FLA, FL-RCA and FL-ConA. Staining of chromatin was seen in several cell types, but was most intense (with each stain) in late erythroblasts. In all malignant cells of the myeloid series there was a general reduction of staining with FLA and an increased binding of FL-ConA at the plasmalemma.

**ABNORMAL SACCHARIDES OF HUMAN LYMPHOID LEUKAEMIAS AND ALLIED LYMPHOMAS.** W. JACOBSON, R. W. STODDART and R. D. COLLINS, *Strangeways Research Laboratory, Cambridge, and Department of Pathology, Vanderbilt University, Nashville, Tennessee, U.S.A.*

Fluorescent-labelled lectins and aprotinin have been used to study the defects in the cell surface and intracellular saccharides of a range of reticuloses. Materials were fixed in anhydrous methanol and used for paraffin sections, or were freshly prepared as spreads. Autofluorescence was eliminated by a short-treatment with osmium tetroxide, before staining. Normal monocytes showed weak, uniform staining for sialic acid and little staining for other sugars. Malignant monocytes stained weakly and irregularly for surface sialic acid and showed very little stain for galactose or mannose; some appeared to show caps. In acute lymphoblastic (human and murine) and chronic lymphocytic leukaemias the malignant cells showed much less staining for sialic acid than their normal counterparts, both at the plasmalemma and nuclear membranes. Staining for mannose was unaltered. There were no differences between B and T lymphocytes. The malignant cells of nodular and thymic lymphomas gave a similar result. In all types of Hodgkin's disease, the neoplastic lymphocytes were characterized as showing a reduction in sialic acid and in sub-terminal galactosyl groups; Reed-Sternberg cells were weakly stained for all sugars. Related, but rather more complex abnormalities were seen in myeloma, Waldenström's macroglobulinaemia and leukaemic reticuloendotheliosis.

**AN ABNORMAL SURFACE PROTEIN OF TUMOUR CELLS.** R. W. STODDART and M. R. PRICE, *Strangeways Research Laboratory, Cambridge, and Cancer Research Campaign Laboratories, University of Nottingham.*

Investigations of a membrane-bound protein of pI 4·00, which is present in the plasma membranes of hepatomas and mammary carcinomas of the rat, have been extended to determine its subcellular location, its relation to the pathology of the tumours, its chemistry and its occurrence in other species. Iodination has shown that the protein is accessible at the surface of hepatoma cells. Traces of a similar protein are present in the nuclear membranes of normal hepatocytes and are greatly elevated in malignancy. In regenerating liver it is maximally elevated at Day 3, but it is far below the level in tumour cells, and does not occur at the plasmalemma. In human, canine, feline, murine and porcine tumours, similar proteins have been found. Foetal tissues (rat and human) contain related proteins of lower pI. The levels of the protein are not related to the histological class of tumour, its invasiveness or antigenicity, its degree of vascularity or the extent of lymphocytic infiltration. There is evidence for its being a glycoprotein. Its appearance during carcinogenesis has been studied.

**THE SYNTHESIS OF α-LACTALBUMIN BY HUMAN MAMMARY CARCINOMAS.** K. L. Woods, D. H. Cove, A. Howell and D. A. Heath, *Department of Medicine, University of Birmingham.*

α-Lactalbumin is the major whey protein of human milk. Using a sensitive radioimmunoassay we have sought evidence for the synthesis of this protein by human mammary carcinomas. The cytosol fraction 14/38 carcinomas contained measurable α-lactalbumin. The presence of α-lactalbumin was closely associated with the presence of oestrogen receptor, and the concentrations of α-lactalbumin and of oestrogen receptor showed a linear correlation. Serum levels of α-lactalbumin were studied in 50 patients with breast cancer and compared with those of healthy control subjects. In normal women, the proportion having detectable circulating α-lactalbumin varied from about 80% in young adults to about 20% in post-menopausal subjects. At all ages the level was generally below 10 ng/ml. The breast-cancer patients showed the same proportion and range of detectable serum levels as age-matched controls. Serum α-lactalbumin was measured prospectively in 100 patients with a variety of breast conditions. Although marked differences were found between patients with benign and malignant breast diseases, these were entirely due to the differeing age structures of the two groups. It is concluded that although synthesis of α-lactalbumin occurs in about a third of human breast carcinomas, assay of this protein in blood is unlikely to help in the diagnosis or management of breast cancer. However, the presence of α-lactalbumin in tumour cytosol is related to the oestrogen-receptor content and may indicate the tumour's hormone responsiveness.

**A COMPARATIVE STUDY OF TWO TUMOUR MARKERS IN BREAST CANCER.** F. Searle, K. D. Bagshawe and G. Goka, *Department of Medical Oncology, Charing Cross Hospital, London.*

Marked serum elevations of carcinoembryonic antigen (Chu and Nemoto (1973) *J. natn. Cancer Inst.*, **51**, 1119), casein (Hendrick and Franchimont (1974) *Europ. J. Cancer*, **10**, 725), and various less specific biochemical markers (Coombes *et al.* (1977) *Lancet*, **i**, 132) have been observed in metastatic breast cancer, but it is necessary to establish whether these markers have a useful role in clinical practice. The serial assay of serum carcinoembyronic antigen in patients undergoing treatment suggests that liver and bone metastases will cause an elevation more readily than does local recurrence. Serum casein is elevated in a proportion of patients with primary breast cancer (24%) of varying histological grade. In a small series of patients who have undergone bilateral mastectomy there is a lower incidence of casein positivity than in a larger series after bilateral mastectomy. Preliminary studies indicate that neither marker is elevated in direct response to certain cytotoxic drug regimens.

**THE COMPETITIVE NATURE OF O⁶-METHYLGUANINE MISCODING DURING DNA SYNTHESIS.** P. J. Abbott and R. Saffhill, *Paterson Laboratories, Christie Hospital and Holt Radium Institute, Manchester.*

The synthetic DNA-like polynucleotide poly(dC-dG) has been methylated *in vitro* with either dimethyl sulphate (DMS) or the potent carcinogen *N*-methyl-*N*-nitrosourea (MNU) and the levels of the various methylation products determined. Treatment with either DMS or MNU resulted in the formation of 3-methylguanine, 7-methylguanine and 3-methylcytosine whilst MNU-methylation also produced $O^6$-methylguanine and phosphotriesters. The methylated polymers were then used as templates for *E. coli* DNA polymerase I in an *in vitro* assay and the amounts of complementary and non-complementary base incorporation measured in concurrent assays. The DMS-methylated polymer did not produce any mis-incorporation, indicating that the products of DMS-methylation do not miscode. The MNU-methylated polymer directed the incorporation of thymine but not of adenine. Presumably this was due to the presence of $O^6$-methylguanine (a promutagenic base) in the template. The thymine incorporation, however, varied with the ratio of the 5′-triphosphates of deoxythymidine and deoxycytidine in the assay, and was less than the $O^6$-methylguanine content of the template. These results indicate that $O^6$-methylguanine is capable of miscoding during DNA synthesis but the miscoding competes with the normal incorporation of cytidine. 3-Methylcytidine, which has been shown to lead to mis-incorporation with RNA polymerase (Ludlum (1971) *Biochim. biophys. Acta*, **247**, 412) does not miscode in our DNA polymerase I system. The competitive nature of the $O^6$-methylguanine miscoding is of interest: presumably it could be another of the many factors determining the tissue specificity of methylating carcinogens.

**CORRECTION OF CHANGES IN LIVER METABOLITES OF MICE FOLLOWING CURATIVE TUMOUR RESECTION.** K. C. Calman, R. A. McAllister and M. Soukop, *Department of Clinical Oncology, Gartnavel General Hospital, Glasgow and Department of Surgery, Western Infirmary, Glasgow.*

Earlier work (Calman and McAllister (1975) *Br. J. Surg.*, **62**, 161; *Br. J. Cancer*, **32**, 247, BACR presentation, Swansea, 31 March 1976), demonstrated in the non-involved liver of mice bearing a TLX-5 lymphoma, C3H mammary tumour, or Sarcoma 180, significant alterations in metabolites, in particular coenzyme A and citrate. Extension of this work has been conducted with the C3H mammary tumour and TLX-5 lymphoma systems. With the C3H mammary tumour, significant depressions ($P < 0{\cdot}001$) of CoA content of liver occurred in the presence of a primary tumour (mean weight 0·5 g). Curative resection of this small tumour caused a return of CoA levels to the normal range. In a second experiment, inoculation of a cell-free supernatant of the TLX-5 lymphoma into normal mice mirrored the metabolic alterations, *i.e.* fall in CoA and a rise in citrate levels which had been seen in tumour-bearing animals. Interestingly, similar increases in spleen weight and concomitant involution of thymus were seen in both groups of mice. In conclusion, further support is given to the suggestion that these changes in liver metabolites are directly related to the presence of a tumour product. However, the nature of this is as yet unknown.

**COMPETITIVE BINDING OF CYCLOPHOSPHAMIDE AND ITS METABOLITES WITH CYCLIC-AMP-BINDING PROTEINS** M. J. Tisdale, *Department of Biochemistry, St. Thomas's Hospital Medical School, London.*

There is a similarity in the biochemical effects of cyclophosphamide and cyclic AMP. Both produce hyperglycemia and cause an increase in tyrosine transamminase, ornithine decarboxylase and alkaline phosphatase activity. This suggests that cyclophosphamide or its metabolites may interact with cyclic-AMP-specific proteins. A 4-hydroxyl substituent in the 1,3,2-oxazaphosphorine ring is required for inhibition of AMP binding to both AMP phosphodiesterase and to the regulatory subunit of the AMP-protein kinase holoenzyme. Binding to the latter causes an activation of the kinase and results in a dissociation into regulatory and catalytic subunits. The inhibitor constant, Ki, for the inhibition of AMP binding to the protein kinase holoenzyme (0·19 mM) correlates well with that for inhibition of the low Km form of the phosphodiesterase. In both cases inhibition is of the competitive type. Although the Ki value for inhibition of phosphodiesterase by 4-hydroxycyclophosphamide is much higher than the $ID_{50}$ value, it causes a time-dependent inactivation of the enzyme probably due to the release of N,N-di(2-chloroethyl)phosphorodiamidic acid. Thus the low affinity binding to phosphodiesterase could act as a highly efficient mechanism for enzyme inhibition. Although 4-ketocyclophosphamide resembles 4-hydroxycyclophosphamide in electron-donating properties, it is inactive with respect to binding to AMP-specific sites. This probably results from the difference in conformation of the rings of these two compounds.

**HYDROXYUREA "SUICIDE" STUDIES ON CLONOGENIC CELLS OF THE LEWIS LUNG CARCINOMA.** A. E. Bateman and G. G. Steel, *Division of Biophysics, Institute of Cancer Research, Sutton, Surrey.*

Studies on cells synchronized *in vitro* have demonstrated that the level of killing by cytotoxic agents varies with the position of the cells in the cell cycle. We present results on Lewis lung tumour clonogenic cells treated *in vivo*, which show variations in survival between S-phase and non-S-phase cells after treatment with cytotoxic drugs. The hydroxyurea suicide technique is used *in vitro* to measure the proportion of clonogenic cells in S-phase both for untreated cells and for cells treated *in vivo* with cyclophosphamide (CY) 1-(2-chloroethyl)-3-cyclohexyl-1-nitrosourea (CCNU) and irradiation. Forty-five per cent of untreated clonogenic cells are in S phase, as thus determined, whereas up to 70% of cells surviving CY and 85% of cells surviving CCNU are in S. We conclude that S-phase cells are more resistant than $G_1$ or $G_2$ cells to these agents.

### O-PHOSPHATE AND O-GLUCURONIDE DERIVATIVES OF *p*-HYDROXYANILINE MUSTARD: POTENTIAL LATENT ANTINEOPLASTIC AGENTS.

P. WORKMAN* and J. A. DOUBLE, *Department of Cancer Research, University of Leeds.*

The O-phosphate (AMPh) and O-glucuronide (AMGl) esters of *p*-hydroxyaniline mustard (AMOH) were synthesised as potential selective agents for tumours containing high levels of phosphatase and β-glucuronidase, respectively. Specificity would be dependent upon their localized conversion to the potently cytotoxic AMOH catalysed by tumour enzymes (Bukhari, Everett and Ross (1971) *Biochem. Pharmac*, **21**, 963). Partitioning studies showed that AMPh and AMGl were more polar than AMOH, due to the presence of the ionized phosphate and glucuronate moieties. The chemical half-life ($t_{1/2}$) of the mustard group of AMGl in aqueous solution (21 min) was longer than that of AMOH (12 min); AMPh ($t_{1/2}$= 13 min) was, however, as reactive as AMOH. Enzyme kinetic studies have shown that AMPh was hydrolysed more rapidly by acid and alkaline phosphatases of mouse bone marrow and small intestinal mucosa than by the corresponding enzymes of transplantable mouse tumours. $K_m$ values for normal and neoplastic mouse tissues were similar. In addition, AMPh was rapidly hydrolysed by blood serum phosphatases. It was thus unlikely that AMPh would be a selective antineoplastic agent. AMPh was, however, more effective than AMOH against transplantable tumours containing comparatively high levels of alkaline phosphatase. A positive correlation was observed between sensitivity to aniline mustard (AM) and tumour β-glucuronidase levels, thus confirming previous findings (Connors and Whisson (1966) *Nature, Lond.*, **210**, 866). Although sensitivity to AMGl also correlated with tumour β-glucuronidase activities this agent was less effective than AM.

* Present address: MRC Clinical Oncology and Radiotherapeutics Unit, The Medical School, Hills Road, Cambridge.

### VIABLE TUMOUR REGIONS INACCESSIBLE TO CHEMOTHERAPEUTIC AGENTS AND A POSSIBLE NEW STRATEGY FOR INACTIVATING THEM.

R. J. GOLDACRE, *Chester Beatty Research Institute, London.*

Studies with systemic dyes have shown that advanced tumours have large ischaemic zones frequently containing substantial quantities of living tumour cells. The question is: are these cells responsible for tumour recurrence after chemotherapy?

Transplantations were made from both the vascular and ischaemic zones (as marked out by systemic dyes) of advanced (9-day) Walker tumours after the rats bearing the tumours had been given chemotherapy by melaphalan at various doses. As the dose increased, the percentage of takes from the vascular zone fell from 100% to zero, whereas the takes from the ischaemic zone remained fairly constant at about 20%.

This shows clearly that chemotherapeutic agents do not reach all stem cells in advanced tumours. Modifying the chemical structure of drugs is unlikely to affect the lack of transport in ischaemic regions, and a new strategy is required for dealing with the inaccessible cells. The following experiments suggest a possible solution.

Advanced Walker tumours after chemotherapy nearly all recurred when left *in situ*, but when *transplanted whole* to new hosts, no tumours grew. However, when the ischaemic zone was transplanted after removing the vascular (killed) shell, many tumours grew. Therefore, the stem cells in the ischaemic zone are unable to penetrate the killed (formerly vascular) shell which has no blood supply since its vessels were cut for the transplantation.

A comparable impenetrable shell was generated *in situ* by a combination of serotonin, which selectively shuts down tumour blood supply, followed after 4 h by melphalan. The serotonin doubled the cure rate, and trebled the survival rate (half life after treatment) of rats bearing advanced Walker tumours.

**SPECIFICITY OF IgG ANTIBODIES IN HODGKIN'S DISEASE.** D. B. Jones, E. V. Elliott,* S. V. Payne and D. H. Wright, *University Department of Pathology and *Tenovus Laboratory, Southampton.*

Hodgkin's spleen tissue cultured for 72 h in the presence of $^{14}$C-leucine shows increased incorporation into secreted IgG measured by a specific immunoprecipitation technique, when compared with controls. IgG prepared by affinity chromatography from the culture supernatant of one patient with a high synthesis rate was capable of binding to human peripheral blood lymphocytes. Angibody capable of binding to human lymphocytes was also present in the serum of this patient and could be typed as IgG K;λ. In a further series of pretreatment Hodgkin's sera screened by Iodinated-protein-A, 20% of patients showed IgG binding to human lymphocytes. However, when further examined on peripheral lymphocyte subpopulations and lymphoid cell lines, the specificity of this antibody was not restricted to T cells as suggested by del Giaco *et al.* (1976) *Biomedicine*, **25**, 79). Further, when tested in a $^{51}$Cr-release assay, none of these sera were able to kill lymphocytes in the presence of complement; preliminary results suggest this lack of cytotoxicity may be due to the subclass of IgG present. Binding sera frequently showed other tissue autoantibody specificities and this suggests that the anti-lymphocyte-antibody present may be an additional disease-associated autoantibody rather than an aetiologic factor associated with a lymphocyte war.

G. S. del Giaco *et al.* (1976). Anti-lymphocyte-antibodies in Systemic Lupus Erythematosus and in Hodgkin's Disease: A Comparison by Immunofluorescence.

**IMMUNOFLUORESCENT STUDIES OF HUMAN-LUNG-CANCER ANTI-SERA.** C. E. Newman, C. H. J. Ford and H. J. Stokes, *University Department of Surgery, Queen Elizabeth Hospital, Birmingham;* G. J. O'Neill, *G.D. Searle Research Laboratories, High Wycombe*; and R. A. Thomson, *Regional Immunology Laboratory, East Birmingham Hospital, Birmingham.*

Thirty xenoantisera have been prepared in goats against lung cancers. Haemaglutinating, haemolytic and lymphocytotoxic antibodies were removed by sequential absorptions with human spleens, and the immunoglobulin (Ig) fractions precipitated. Each absorbed Ig was examined for selective uptake by the tumour cells against which it was prepared. The test system is an indirect immunofluorescent (IF) test using cryostat-cut sections from specimens of the original tumour, snap frozen and stored in liquid $N_2$, and a rabbit anti-goat gammaglobulin fluorescein-isothiocyanate conjugate. Every Ig showed selective localization by the original tumour cells. Titres ranged from neat to 1/32. In most cases, non-specific fluorescence was observed against connective tissue and endothelial cells. This was always similar to that seen with an Ig prepared in the same way against a mycosis fungoides tumour. Significant selective localization of this Ig by lung tumour cells was not observed. Absorbed Igs showed positive tumour-cell fluorescence when examined for selective localisation by lung tumours of the same and different histological types, suggesting a surprisingly high cross-reactivity. A xenoantiserum prepared against cultured oat-cell carcinoma cells had high titres of haemolytic (1/96) haemagglutinating (1/512) and lymphocytotoxic (1/3072) antibodies which were removed by absorption. After absorption, the IF titre against lung tumour cells was 1/1200. The fractionated Ig has an IF titre of 1/80 to 1/160. This reagent has been carefully assessed against a panel of 8 lung cancer sections comprising 2 of each of the histological groups viz. oat-cell, anaplastic, adeno and squamous. In 7/8, the tumour-cell concentration of IF-detected antibody is apparent at titres of 1/80 to 1/160. The control Ig (mycosis fungoides) does not demonstrate this tumour-cell concentration. This evidence suggests the selective localization of tumour-cell antigens on human lung cancer cells. These may be tumour-specific, tumour-associated or even normal cellular antigens selectively concentrated in the tumour-cell membrane.

**THE DEMONSTRATION OF DEPRESSED LEVELS OF T LYMPHOCYTES IN BREAST CANCER PATIENTS IS DEPENDENT ON THE METHOD USED.** R. H. WHITEHEAD, G. P. ROBERTS, J. THATCHER and L. E. HUGHES, *University Department of Surgery, Welsh National School of Medicine.*

There are a number of conflicting reports on the proportion of E-rosetting cells (T lymphocytes) detectable in patients with breast cancer. However, different rosetting techniques have been used in each study, and it was felt that this might be the cause of the differing results obtained. We have therefore compared three standard rosetting techniques:

(a) short incubation period of $1\frac{1}{2}$ h at 4 °C in PBS

(b) overnight incubation at 4 °C in PBS and

(c) $1\frac{1}{2}$-h incubation at 4 °C in 5% FCS.

In addition, the effect of methodology on cancer-serum-induced inhibition of E-rosette formation by normal lymphocytes has been studied. It was found that when the number of E-rosetting cells was determined using incubation at 4 °C for $1\frac{1}{2}$ h, levels in women with breast cancer and an age-matched control group were both significantly lower than the levels obtained for a young control group. There was no difference between the 3 groups when rosetting was performed in 5% FCS or by overnight incubation, as the proportion of E-rosetting cells was then higher in the first two groups. Inhibition of E-rosette formation by incubating normal lymphocytes in breast cancer serum could be demonstrated using a short incubation period ($1\frac{1}{2}$ h) but not after overnight incubation or after incubating the treated lymphocytes in saline overnight at 4 °C before rosetting. These findings explain the previous conflicting results, and suggest the presence of a factor(s) on the surface of T lymphocytes of cancer patients and in the sera of these patients which binds reversibly to the lymphocyte surface and in some way masks the E-receptor site.

**A TWO-STAGE ASSAY FOR TUMOUR-DIRECTED CELL-MEDIATED IMMUNITY.** A. J. COCHRAN, R. M. MACKIE, L. J. OGG, A. M. JACKSON, C. E. ROSS and G. TODD, *University Departments of Pathology and Dermatology, The Western Infirmary, Glasgow.*

In an attempt to overcome problems of the one-stage leucocyte migration inhibition assay we are investigating a two-stage test. In Stage I Ficoll-Hypaque (FH) separated mononuclear cells are incubated with formalin-fixed cells (FC) of the appropriate tumour type, of other tumour types and with formalinized normal cells. After 24 h the migration-inhibiting activity of the various supernatants is assessed relative to the supernatant of FH cells incubated in the absence of formalinized cells. The indicator-cell population is gravity-sedimented peripheral blood leucocytes from a normal individual. Active supernatants resulted more often from co-cultre of melanoma leucocytes (ML) with melanoma cells (17/39–44%) than from cultures of ML with other types of cells (6/41–15%). Active supernatants infrequently resulted from culture of normal FH cells with FC (control FH cells/melanoma FC, 1/30 (3%) control FH cells/other FC, 4/22 (18%). Active supernatants were most frequent with FH cells from Stage II melanoma patients (Stage I, 1/6 (17%), Stage II, 14/25 (56%) and Stage III, 2/8 (25%). In combinations of ML with melanoma FC the reaction frequency increased with the number of FC preparations tested. This was not seen with combinations of ML and other FC or control leucocyte cultures with melanoma or other FC. The direct and two-stage assays were concordant in about 70% of concurrent tests.

**A CONTROLLED TRIAL OF ACTIVE IMMUNOTHERAPY IN THE MANAGEMENT OF STAGE IIB MALIGNANT MELANOMA.** M. B. MCILLMURRAY, M. J. EMBLETON, W. G. REEVES, M. J. S. LANGMAN and M. DEANE,* *Department of Therapeutics, Cancer Research Campaign Laboratories, Department of Immunology, University of Nottingham and *the Plastic Unit, City Hospital, Nottingham.*

Within two years of operation about three-quarters of all patients with malignant

| | Recurrence rate | | Death rate | |
|---|---|---|---|---|
| | Vaccinated | Control | Vaccinated | Control |
| 3 months | 3/8 | 0/7 | 2/8 | 0/7 |
| 6 months | 4/8 | 2/7 | 3/8 | 0/7 |
| 12 months | 4/8 | 4/6 | 4/8 | 0/6 |

melanoma who have regional nodes involved (Stage IIb) will have obvious recurrences and more than half of these will have died (Lane, N., Lattes, R. and Malm, J. (1958) *Cancer*, **11**, 1025. Improvements in results are therefore only likely to ensue if some other treatment can be added to excision. Animal studies indicate that host resistance to transplantable tumours is enhanced when BCG and tumour-cell vaccines are given together as contact immunotherapy, provided that the tumour load is small, suggesting that such a combination may be a more effective immunostimulant than either given alone (Bast, R. C. *et al.* (1974) *New Engl. J. Med.* **290**, 1413) Fifteen patients with Stage IIb malignant melanoma were randomly allocated to either a treatment group in which they received a vaccine ($3 \times 10^7$ live BCG and $5 \times 10^7$ autologous irradiated tumour cells) or a control group who received no further treatment after surgical eradication of their disease. The recurrence and death rates in the two groups one year later are shown in the table.

These results suggest that active immunotherapy may cause tumour enhancement in some patients with malignant melanoma, and that claims of benefit from uncontrolled and unrandomized studies should not be readily accepted.

**IMMUNOLOGICAL MONITORING OF PATIENTS UNDERGOING ACTIVE IMMUNOTHERAPY FOR STAGE IIB MALIGNANT MELANOMA.** M. J. Embleton, M. B. McIllmurray, J. H. Ransom and W. G. Reeves, *Cancer Research Campaign Laboratories and Departments of Therapeutics and Immunology, University of Nottingham.*

Fifteen patients with Stage IIB malignant melanoma were allocated to a group receiving a vaccine ($3 \times 10^7$ live BCG and $5 \times 10^7$ autologous irradiated tumour cells) following tumour resection, and a control group who were given no further treatment. All patients were monitored immunologically before treatment, and at 3, 6 and 12 months after treatment, in order to look for changes in immune function which might correlate with their clinical course, and thus provide a test with prognostic significance. Immunocompetence of patients was assessed by skin tests, using the recall antigens PPD and Varidase, measurement of blood components, and by lymphocyte transformation *in vitro* with various agents. Attempts were made to evaluate tumour-directed immunity using *in vitro* tests for both leucocyte-mediated and antibody-mediated activity against melanoma extracts or cultured cells. There was a trend towards earlier tumour recurrences in patients with poor skin reactivity to PPD and Varidase at the beginning of treatment. The *in vitro* measurements fluctuated throughout the time course for each patient, and none revealed any significant differences between patients with good or bad prognosis, or between vaccinated and control patients. It is concluded that *in vitro* monitoring using present techniques is of no practical value in prognosis of malignant melanoma.

**RESULTS, FOR 27 MONTHS OF FOLLOW-UP, OF A STRATIFIED RANDOMIZED TRIAL OF INTRADERMAL BCG IN ADDITION TO CONVENTIONAL TREATMENT IN PATIENTS WITH LUNG CANCER.** H. M. Anthony, K. E. Madsen, M. K. Mason and G. H. Templeman, *University Department of Immunology, Leeds General Infirmary and Killingbeck Hospital, Leeds.*

Random allocation of 75 men with confirmed bronchial carcinoma to BCG (0·1 ml Glaxo BCG i.d. monthly to 6 months) or control within a stratification system based on conventional therapy and other prognostic factors, showed no significant prolongation of life by BCG, by sequential analysis of 23 pairs or by life table analysis (computer program kindly loaned by Dr P. G. Smith of Oxford). The latter analysis showed significant prolongation of survival in "acceptable clinical" condition ($P = 0 \cdot 0049$) or good general condition ($P = 0 \cdot 0112$) for BCG-treated radical radiotherapy patients. For almost all groups, BCG had greater effect on prolongation of "acceptable clinical" or good general condition than on survival to death. BCG also reduced weight loss. These effects could be due to increase in T-lymphocyte proportion by BCG (Anthony *et al.* (1975) *Clin. exp. Immunol.*, **20**, 40) or to stimulation of macrophages. In patients with squamous carcinoma, peripheral blood lymphocytes and monocytes directly correlated with length of survival ($P < 0 \cdot 02$, $P < 0 \cdot 04$) in keeping with partial immune control. For oat cell carcinoma, lymphocyte numbers *inversely* correlated with "Survival" ($P < 0 \cdot 04$) as did the trend with monocyte number ($P < 0 \cdot 03$) suggesting resistance to immune cytolysis in oat cell carcinomas and a stimulating effect for immune attack.

**C-TYPE RNA TUMOUR VIRUSES: ISOLATION AND CHARACTERIZATION OF A COMPLETE DNA COPY OF THE ERYTHROID-SPECIFIC FRIEND VIRUS GENOME.** I. B. Pragnell, W. Ostertag* and J. Paul, *Beatson Institute for Cancer Research, Wolfson Laboratory for Molecular Pathology, Glasgow and *Max Planck Institut fur Exp. Med., Göttingen, West Germany.*

Friend virus (FV) is a C-type RNA tumour virus which induces an erythroleukaemia in susceptible mice, and the transformed erythroid cells can be maintained in tissue culture (Friend *et al.* (1966) *Nat. Canc. Monogr.*, **22**, 505). These cells can be stimulated to differentiate along the erythroid pathway by addition of aprotonic solvents such as dimethylsulphoxide (Friend *et al.* (1971) *Proc. Nat. Acad. Sci., U.S.A.*, **68**, 378; Ostertag *et al.* (1972) *Nature, New Biol.*, **243**, 203). We have synthesized and characterized a viral $^3H$-labelled complementary DNA (cDNA) derived from the Friend virus genome. Hybridization analysis has shown that:

(a) The FV cDNA is a full-length copy of the Friend virus genome.
(b) The base-sequence complexity of the viral genome is $4 \times 10^6$ daltons.
(c) There are 4–7 viral genes homologous to the FV genome in normal (DBA/2) mouse DNA and 10–12 viral genes per haploid genome in DNA from the FV-transformed Friend cell. A significant minor proportion (20–30%) of the FV cDNA probe anneals only to virus related sequences in the transformed-cell DNA, indicating that additional FV-related sequences are integrated in the transformed-cell DNA. The Friend virus consists of a helper lymphatic leukaemia virus (LLV) and the erythroid-specific defective spleen-focus-forming virus (SFFV). We have isolated by end-point dilution and cloned a cell line producing only the LLV component. The 70S RNA from LLV can be used to remove the LLV sequences from FV cDNA, resulting in enrichment of the erythroid-specific sequences and/or the sequences involved in transformation of the target cell.

**CELL-MEDIATED RESPONSE TO SIMIAN ONCORNAVIRUSES IN WOMEN DURING PREGNANCY.** L. Thiry, S. Sprecher-Goldberger, M. Bossens and F. Neuray, *Institut Pasteur du Brabant and Free University of Brussels.* (*Introduced by F. J. Lejeune*).

Baboon type-C virus and Mason-Pfizer virus (MPV) were added to short term human leucocyte cultures and induced a high level of thymidine incorporation, due to virus replication. Killed viruses caused a limited but significant level of thymidine incorporation in some leucocyte cultures, indicating that some individuals possess lymphocytes sensitized to antigens carried by one of the viruses. Cells chronically infected with each virus, or not infected, were treated with mitomycin C; one type of infected culture specifically stimulated some leucocyte cultures, but responses to the infected cells were not always associated with responses to the corresponding virus. Because oncornavirus particles have been described in placentas, lymphocyte responses to the Baboon virus and to MPV were studied in 30 women at the end of pregnancy and in 37 non pregant women. Lymphocyte responses to *cells* infected with either the Baboon virus or with MPV were found in 36% and 2·6% of the pregnant and non-pregnant women, respectively, and were most frequent in women with many gestations. The number of responses to one of the two virus *particles* was not different in pregnant and non-pregnant women, but increased with the number of gestations, since they were found in 0% of gravida 0, in 16% of women with 1–4 gestations, and in 53% of women with 5–7 gestations. Antigens similar to those of Baboon virus or MPV may be expressed during gestations.

**VSV PSEUDOTYPES PRODUCED IN HUMAN MELANOMA CELL LINES.** N. VAN TIEGHEM, D. LITEANU, A. F. VERCAMMEN-GRANDJEAN, P. VANDENBUSSCHE, D. DEKEGEL, L. BEAUMONT and F. J. LEJEUNE, *Université Libre de Bruxelles, Institut Pasteur du Brabant and Institut Bordet, Bruxelles.*

Three human melanoma sublines were investigated for viral particles. Electron microscope studies showed a high production of melanosomes and viral particles budding into the cisternae of the endoplasmic reticulum in cells derived from a subcutaneous metastasis (HM6B-A). This viral expression was related to melanin expression, and could be switched on or off by adding to, or subtracting tyrosine from the culture media. When infecting an amelanotic subline (HM6B-N), provided by the same patient, with a VSV thermolabile mutant (tl) there was production of a VSV pseudotype (Zavada, J. (1972) *Nature, New Biol.*, **240**, 124). The coat of progeny VSV was modified: the thermolabile virus had become thermostable. This cell line did not show virus expression either after the tyrosine test or after treatment with halogenated pyrimidines (Lowy, D. R., Rowe, W. P., Teich, N. and Hartley, J. W. (1971) *Science*, **174**, 155). The amount of VSV pseudotype particles was increased 2500× in the presence of (5-IUdR). Reverse transcriptase activity in the culture supernatant was barely detectable. The VSV pseudotypes could be neutralized by the patient's serum. When treating another amelanotic subline (HM6A) from the same origin with DL-DOPA ($8{\cdot}0 \times 10^{-5}$ M/ml) it was possible to detect a few virus-like particles. Thermostable pseudotypes were also obtained in the presence of this drug. Thus, VSV pseudotype particles could be used as a tool to detect one (or more) neutralization antigens to one (or more) "putative" human melanomaviruses.

**CROSS-REACTIVITY OF ANTISERA TO ONCOGENIC RNA VIRUS PROTEINS WITH HUMAN LEUKAEMIA CELLS.** A. PILLAI,*, N. HOGG† and R. T. D. OLIVER, **Imperial Cancer Research Fund, Department of Medical Oncology, St Bartholomew's Hospital, London, and †Imperial Cancer Research Fund, Tumour Immunology Unit, University College, London.*

Antisera raised in rabbits against PAGE-separated proteins from disrupted Moloney virus have been tested against a panel of leukaemia cells from 17 patients with AML, 6 patients with ALL, 5 patients with CLL, 7 remission lymphocytes from 7 patients with acute leukaemia and lymphocytes from 8 normal laboratory controls. A standard microcytotoxicity assay with absorbed weanling rabbit serum as a complement source was used. Reactivity was greatest in serum against gp 79/80 (18/28 positive with from 12–80% cytotoxicity) and least with serum against p30 (6/28 positive with from 10–50% cytotoxicity). Intermediate reactivity was observed with antisera to p15 (envelope), p15 and p12. Reactivity against remission lymphocytes was considerably less than against leukaemia cells, although 3/15 cells tested did show slight reactivity (12–42%). Cross-absorption experiments with different types of leukaemia cells suggest that the determinants detected on ALL and CLL cells are the same as on AML. Studies are in progress, using the lysostrip technique, to clarify the relationship of the determinants detected by these sera to normal tissue antigens, $B_2$ microglobulin and HLA.

**EFFECT OF HYPERTHERMIA ON THE IMMUNOCOMPETENCE OF NORMAL AND VX2 TUMOUR-BEARING RABBITS.** S. A. SHAH and J. A. DICKSON, *Cancer Research Unit, University Department of Clinical Biochemistry, Royal Victoria Infirmary, Newcastle-upon-Tyne.*

Following effective heat treatment of a primary cancer in man and in animals, tumour metastases also disappear with cure of the host. With the rabbit VX2 carcinoma, heat applied locally to the tumour is more effective than total-body heating, and it is believed that an altered response of the animal's immune system may be involved in this difference (Dickson (1976) *Int. Symp. Cancer Therapy by Hyperthermia and Radiation.* Am. Coll. Radiology Press, Baltimore, Md. p. 134). In the present study, 9/12 VX2 tumour (15–20 ml) bearing rabbits treated by Local Radiofrequency Heating (LRFH, 47–50 °C/30 min), and 1/8 rabbits treated by LRFH followed 8 days later by Total-body Hyperthermia (TBH) at 42 °C (1 h on each of 3 successive days) were cured. Skin response to challenge with Dinitrochlorobenzene (DNCB) or tumour extracts, and the anamnestic response to bovine serum albumin (BSA) in tumour bearing rabbits increased following LRFH with untreated or (LRFH + TBH) treated rabbits. *In vitro*, lymphocytes plus serum from cured animals caused greater inhibition (30–65%) of tumour cells than lymphocytes plus serum from untreated animals (20–30%). In normal rabbits, LRFH or TBH did not affect the skin response to DNCB. The response to BSA was reduced by up to 1/25 normal level following LRFH and by up to 1/80 normal level after TBH. The data support the previous postulate that TBH suppresses the immune system.

### CELL-MEDIATED CYTOTOXICITY IN TUMOUR-BEARING DOGS.

G. R. Betton, *Oncology Unit, Department of Clinical Veterinary Medicine, University of Cambridge.*

Dogs bearing spontaneous neoplasms were tested for peripheral blood lymphocytotoxicity using $^{51}$Cr-labelled allogeneic tumour target cells. Where possible, sequential testing was performed during the course of therapy. Analysis of results obtained with melanoma-, osteosarcoma- and mammary-carcinoma-bearer peripheral blood lymphocyte preparations tested against tumour target cells of the same and different histological types showed no evidence for type specificity. Healthy control donors also exhibited non-specific cytotoxicity in a proportion of donors, that was not significantly different from that observed in tumour bearers. Sequential testing of tumour bearers showed no consistent responses to treatments such as i.v. BCG immunotherapy, but dogs involved with metastatic disease showed lower responses. Responses of BCG-treated tumour bearers were lower than those seen in healthy BCG-treated controls. The use of autochthonous tumour target cells was unsatisfactory, as short-term cell cultures were resistant to lysis in the $^{51}$Cr-release assay when compared with established allogeneic cell lines. The non-specific effector cell rosetted with human erythrocytes, a canine T-cell marker, but carbonyl iron treatment also reduced non-specific cytotoxicity in some cases. Certain target cell lines, e.g. osteosarcoma, were particularly sensitive to non-specific lysis. Lymphocytotoxicity detected in the allogeneic $^{51}$Cr release assay was therefore not directed at tumour specific antigens and was present also in healthy controls. Spontaneous canine neoplasms appeared to lack histological-type-specific antigens capable of eliciting a cell-mediated cytotoxic response in the tumour-bearing host.

### STUDIES ON THE MICROCYTOTOXICITY TEST: THE UPTAKE OF AMINO ACIDS BUT NOT NUCLEOSIDES PROVIDES A DIRECT AND QUANTITATIVE MEASURE OF TARGET CELL SURVIVAL.

R. C. Rees and C. G. Brooks, *Cancer Research Campaign Laboratories, University of Nottingham.*

Optimal labelling of tumour cells with radionucleosides required that these precursors be present at high concentration, because many tumour-cell targets did not utilize exogenous nucleoside efficiently when present at low concentration. However, even using relatively high concentrations of radionucleoside, large discrepancies between radionucleoside uptake and cell survival assessed by cell counting were often found. Analysis revealed that two types of soluble factors released by lymphoid cells were responsible for the discrepancies.

(a) Competitive inhibitors of nucleoside uptake, removable by washing.

(b) Factors which caused an irreversible disruption of tumour cell nucleoside metabolism without any apparent effect on cell survival.

In contrast to the severe problems encountered with radionucleosides, radio-labelled amino acids were taken up equally avidly by all tumour cells tested, and provided a direct and precise measure of target-cell survival, because neither competitive nor non-competitive interference with amino-acid uptake caused by lymphocytes or lymphocyte factors was detectable. The use of the $\gamma$-emitting $^{75}$Se-methionine as precursor permitted a simple and rapid method of quantitating target-cell survival in the microcytotoxicity test.

### SPONTANEOUS DEVELOPMENT OF CYTOTOXIC ACTIVITY IN CULTURES OF LYMPH NODE CELLS FROM TUMOUR-BEARING RATS.

R. A. ROBINS, *Cancer Research Campaign Laboratories, University of Nottingham.*

During *in vitro* experiments attempting to induce cellular cytotoxicity by syngeneic lymphocytes to chemically induced rat tumours, and to boost the cytotoxicity of lymph node cells from rats exposed to tumour *in vivo*, it was observed that lymph node cells from rats bearing a transplanted methylcholanthrene-induced sarcoma became highly cytotoxic when cultured without addition of tumour antigen. This cytotoxicity could be very strong: significant reduction in target-cell survival was observed with 100 effector cells per well in a $^{125}$IUdR post-label microcytotoxicity test (effector:target ratio of 1:5); at E:T ratios of 10:1, over 90% cytotoxicity was obtained. In contrast, cultured lymph node cells from normal rats only showed low levels of cytotoxicity at relatively high E:T ratios. Cultured lymph node cells from tumour-bearing and normal rats were also tested in a $^{51}$Cr-release assay. Cytotoxicity was not detected in this assay, even with a 17-h incubation period and high E:T ratios using lymphocyte preparations shown to be highly cytotoxic in a 24–48-h post-label microcytotoxicity test. At least 4 days of culture were necessary for augmented cytotoxicity to be detected, but cytotoxicity did not increase further between 4 and 7 days of culture. Yields of lymphocytes were normally between 40 and 50% after 7 days. Cultured lymphocytes could be stored in liquid $N_2$ after programmed freezing, and almost full cytotoxic activity recovered after thawing. These experiments show that potent cytotoxic activity can be generated in lymphocyte culture, and that some target-cell systems may require a microcytotoxicity test to detect cytotoxicity. The exact culture requirements for the development of cytoxicity and the nature and mechanisms of its effector phase are at present under investigation.

### THE USE OF A $^{51}$Cr-RELEASE TEST FOR THE DETECTION OF COMPLEMENT-DEPENDENT CYTOTOXICITY OF RAT HEPATOMA-BEARER SERUM.

M. R. PRICE, *Cancer Research Campaign Laboratories, University of Nottingham.*

A short-term $^{51}$Cr-release test was developed for the detection of complement-dependent cytolytic activity of sera from donors bearing an aminoazo-dye-induced rat hepatoma for transplanted tumour cells. Cytotoxicity was evident in the serum of donors bearing i.p. implants of hepatoma, but not in sera from animals bearing s.c. transplants or immunized with $\gamma$-irradiated tumour tissue. Sera from syngeneic multiparous donors sensitized to tumour-associated embryonic antigens, also failed to exhibit a cytotoxic response against tumour cells. Cytotoxic tumour-bearer sera displayed individually distinct, tumour-specific reactivity against hepatoma target cells which, with selected sera, was still detectable at final dilutions of 1/100. Although these sera contained tumour-specific IgG antibody demonstrable using the indirect membrane-immunofluorescence test, cytolytic activity fractionated in the 19*s* region of Sephadex G200 gel-filtration column eluates. This reactivity was removed by absorption with cells of the same hepatoma as that borne by serum donors, whereas absorption with cells of other hepatomas was without effect. The test developed is both objective and reproducible and, used in conjunction with the characterized syngeneic serological reagents available, it should prove of value for the quantitation of tumour antigens associated with chemically induced rat hepatomas.

**ANTIGENIC HETEROGENEITY WITHIN PRIMARY 3-METHYLCHOLANTHRENE-INDUCED RAT SARCOMAS.** M. V. PIMM, *Cancer Research Campaign Laboratories, University of Nottingham.*

Studies by Prehn (1970) *J. natn. Cancer Inst.* **45**, 1039, with primary 3-methylcholanthrene (Mc)-induced mouse sarcomas have demonstrated the possibility of antigenic heterogeneity within individual established tumours, so that a transplant line initiated with tissue from one part of a primary sarcoma was occasionally antigenically distinct from a line established from another part of the same tumour. In the present study the immunogenicities of *in vivo* lines established from primary Mc-induced rat sarcomas have been compared with those of lines initiated from tumour recurrences at the site of the primaries' surgical excisions. Lines from 2/4 primary sarcomas showed little or no immunogenicity, as assessed by protection to challenge afforded by graft excision or implantation of irradiated tissue. In contrast, lines from all 4 recurrences were immunogenic, giving protection against up to $5 \times 10^6$ tumour cells. Most importantly, with all 4 tumours, lines established from recurrences were antigenically distinct from lines from their original primary sarcomas, so that immunization with regrowth lines gave no protection to the lines from the primaries, and *vice versa*. These studies support the concept that primary Mc-induced tumours may be antigenically heterogeneous, and demonstrate that outgrowth of a second, antigenically distinct, tumour follows surgical removal of the primary. These findings also have implications for the design of immunotherapy protocols for recurrences or metastases from experimental or even human tumours.

**ULTRASTRUCTURAL STUDIES OF INTERACTIONS BETWEEN HOST INFLAMMATORY CELLS AND TUMOUR CELLS WITHIN TRANSPLANTABLE HAMSTER FIBROSARCOMAS.** R. G. P. PUGH-HUMPHREYS, *Experimental Pathology Unit, Department of Zoology, Aberdeen University.*

Transplantable malignant fibrosarcomas, produced initially by s.c. injection of syngeneic hamsters with polyoma-virus-transformed BHK 21/C13 fibroblasts, grew rapidly and invaded adjacent host muscle and connective tissue. Host inflammatory cells were observed, dispersed singly and in groups within tumour tissue, and these cells appeared to mediate focal necrosis of tumour cells which could not be accounted for simply by lack of tumour vascularization or coagulation necrosis. Ultrastructural studies revealed the presence of plasma cells and aggregates of lymphocytes and phagocytes among the tumour cells, which are indicative of a host immune response against the tumour (Moore, Nisbet and Haigh (1973) *Br. J. Cancer*, **28**, Suppl. 1). Ultrastructural examination of contacts between lymphocytes and tumour cells did not reveal the presence of any specialized intercellular junctions although protrusion of lymphocyte pseudopodia into tumour cells and tumour-cell lysis were observed. Endocytosis and destruction of tumour cells by polymorphonuclear leucocytes (PMNs) and mononuclear phagocytes were observed; whereas PMNs engaged in microphagocytosis (i.e. phagocytosis of small portions of tumour cells) the mononuclear phagocytes attempted to engulf entire cells. The mononuclear phagocytes had the ultrastructural appearance of "activated macrophages" (Carr (1973) *The Macrophage: a review of ultrastructure and function*, Acad. Press) and appeared to mediate much of the tumour fibroblast destruction as observed in other tumour systems (Evans (1973) *Br. J. Cancer*, **28**, Suppl. 1, 19). Accumulation of intercellular material around many of the tumour fibroblasts appeared to afford these cells protection from direct contact and attack by host inflammatory cells.

**ALVEOLAR MACROPHAGE CYTOTOXICITY IN THE DOG.** N. T. GORMAN, *Oncology Unit, School of Veterinary Clinical Studies, Cambridge University.*

The cytotoxicity of alveolar macrophages from a total of 33 dogs has been examined using the $^{51}Cr$-release assay with allogeneic long-term tissue-culture cells as targets. It has been found that alveolar macrophages from unstimulated dogs (8) do not exhibit any cytotoxicity. However, in the case of those animals which received i.v. BCG (10) and developed diffuse granulomatous lesions of the lung, the cytotoxicity was marked; this was not found in 3 dogs which had received intrathoracic BCG. The observed cytotoxicity appeared to be non-specific, with a lack of selectivity between cells of neoplastic origin or normal canine kidney at the ratios examined (10:1, 20:1, 40:1). An attempt has been made to specifically immunize 9 dogs against allogeneic tumour cells in one of the following ways:

(a) 3 i.v. injections of $5 \times 10^8$ cells

(b) 3 i.v.injections of $5 \times 10^8$ cells plus 3 mg BCG (Glaxo Laboratories)

(c) 3 i.v. injections of $5 \times 10^8$ cells sonicated with Freund's adjuvant plus 0·5 mg heat-killed BCG.

In these experiments it was found that only dogs which received i.v. BCG demonstrated any cytotoxicity. This however, lacked specificity for the immunizing cell. In a further series of 3 dogs which received more immunizations with Freund's adjuvant, cells and heat-killed BCG, non-specific cytotoxicity has been observed. Examination of the supernatants of cultures of alveolar macrophage from both normal dogs and those which had received i.v. BCG failed to reveal any soluble factor which could produce the observed cytotoxicity.

### EFFECTS OF CORTISONE ACETATE AND SPARINE ON THE PRIMARY LEWIS LUNG CARCINOMA AND ITS PULMONARY METASTASES AND ON THE ACTION OF *C. PARVUM*.

T. E. Sadler, P. D. E. Jones, H. D. Mitcheson and J. E. Castro, *Urology and Transplantation Unit, Royal Postgraduate Medical School, Hammersmith Hospital, London.*

*Corynebacterium parvum* inhibits the growth of a variety of animal tumours and it is now undergoing clinical trials. In man, this vaccine causes undesirable side-effects, including nausea and pyrexia. Hydrocortisone and sparine have been used to relieve these symptoms. In this study, the effects of cortisone acetate (CA) or sparine on the growth of the primary Lewis lung carcinoma and its pulmonary metastases and the action of *C. parvum* were investigated in C57BL mice.

A high dose of CA similar to that used by Fisher *et al.* (1976) *J. natn. Cancer Inst.*, **56**, 571, had little effect on primary tumour growth but significantly *enhanced* metastases. I.v. *C. parvum* given to these mice before or after CA treatment caused no further tumour inhibition, but significantly reduced metastases. However, the number of metastases in mice which were given combined *C. parvum* and CA was not significantly different from that found in control mice, and significantly greater than that found in mice which received only *C. parvum*. Thus CA effectively counteracted the beneficial antimetastatic action of *C. parvum*. A low dose of CA (human equivalent) or sparine did not alter primary tumour growth or the antimetastatic action of *C. parvum*.

We conclude that all drugs being given to patients to counteract the side effects of immunotherapy should be examined experimentally to determine their effect on both primary and secondary tumours.

### THE MECHANISM OF THE ANTI-TUMOUR EFFECT OF GLUCANS AND FRUCTOSANS. A COMPARISON WITH *C. PARVUM*.

R. Bomford & C. Moreno, *Department of Experimental Immunobiology, Wellcome Research Laboratories, Beckenham, Kent.*

The antitumour activity induced by glucans (lentinan, yeast cell walls, pseudonigeran, dextran, DEAE-dextran and dextran sulphate) and fructosans (levan and carboxymethyl-levan) was compared with the activity of *C. parvum*. The following effects on tumour systems in CBA mice were assayed: (a) adjuvant activity on the immune response against tumour-specific transplantation antigens (TSTA) with a methylcholanthrene-induced fibrosarcoma; (b) cytostatic activity of peritoneal macrophages against radiation-induced leukaemia cells; and (c) inhibition of nodule formation in the lungs following i.v. injection of fibrosarcoma cells.

All the polysaccharides induced cytostatic macrophages, but the dextrans and levans did so only after i.p. and not i.v. injection. Only lentinan, yeast cell walls and pseudonigeran were active in the lung-nodule-inhibition test; and only lentinan and dextran sulphate showed slight adjuvant activity for TSTA. It is concluded that the antitumour activity induced by these polysaccharides is predominantly non-specific macrophage-mediated and much weaker than that found with *C. parvum*.

**SYNERGISTIC COMBINATION OF CHEMO- AND IMMUNO-THERAPY IN A MOUSE TUMOUR SYSTEM.** M. T. SCOTT, *Department of Experimental Immunobiology, Wellcome Research Laboratories, Beckenham, Kent.*

Treatment of a chemically induced mouse solid fibrosarcoma, using either non-specific (*C. parvum* 350 μg i.v.) or specific active (s.c. *C. parvum* mixed with $5 \times 10^5$ irradiated tumour cells) immunotherapy, 4 days after a single dose of cyclophosphamide (200 mg/kg) was synergistically more effective than either *C. parvum* or drug treatment alone. A contributory factor may be that cyclophosphamide pretreatment has been shown to potentiate the specific antitumour immunity that arises from *C. parvum* interaction with tumour antigen. Systemic *C. parvum* before cyclophosphamide will potentiate the antitumour effects of the drug; previously ineffective low doses becoming effective. No similar potentiation of the effects of another alkylating agent, Melphalan, was evident.

**ENHANCEMENT OF THE ANTITUMOUR EFFECTIVENESS OF METHOTREXATE THROUGH SELECTIVE PROTECTION OF NORMAL TISSUES.** G. A. TAYLOR, G. P. BROWMAN and K. R. HARRAP, *Department of Biochemical Pharmacology, Institute of Cancer Research, Sutton, Surrey.*

The use of folinic acid (citrovorum factor) to limit the toxicity of methotrexate (MTX) to normal proliferating tissues, is an established clinical procedure during intensive MTX therapy ((1975) *Cancer Chemother. Rep.*, **6**). In previous reports we have shown that MTX induces a purineless state in bone marrow and small intestine when administered to mice (Talbot *et al.* (1976) *Br. J. Cancer*, **34**, 321; Straw *et al.* (1977) *J. natn. Cancer Inst.*, **58**, 91), the deficiency being established earlier in the gut than in bone marrow. We have currently been investigating the possibility that normal proliferating tissues of the mouse can be protected from MTX toxicity by the administration of purine and pyrimidine nucleosides or bases. It was demonstrated that a pyrimidine alone could not protect from the toxic effects of MTX. However, combinations of thymidine (TdR) and hypoxanthine (Hx) (together with allopurinol (Ap)) do protect the mouse from MTX toxicity, with an efficacy comparable to folinic acid. Furthermore, rescue of L1210-tumour-bearing animals with TdR/Hx/Ap combinations, following MTX treatment, can produce a median survival in excess of that achieved with folinic-acid rescue. It would seem that purine/pyrimidine rescue techniques may exploit selective differences in salvage-pathway utilization between tumour and normal tissues, and may have clinical application.

**DIHYDROFOLATE-REDUCTASE ACTIVITY IN MOUSE GUT AFTER TREATMENT WITH METHOTREXATE, AND RESCUE WITH VARIOUS AGENTS.** A. H. CALVERT and K. R. HARRAP, *Department of Biochemical Pharmacology, Institute of Cancer Research, Sutton, Surrey.*

Recently, considerable attention has been paid to the selective protection of normal tissues from the effects of methotrexate (MTX) by the use of purines and pyrimidines, with the object of increasing the therapeutic index of this drug (Straw *et al.* (1977) *J. natn. Cancer Inst.*, **58**, 91). The toxicity of MTX is dependent on the time for which a 95% inhibition of dihydrofolate reductase (DHFR) is maintained (Goldie *et al.* (1972) *Eur. J. Cancer*, **8**, 409; Jackson and Harrap (1973) *Arch. Biochem. Biophys.*, **158**, 2). Therefore it is important to assess this parameter in addition to a study of the total plasma and tissue levels of MTX in those animal models used for testing rescue protocols. A technique has been developed for measuring the *in vivo* inhibition of DHFR, making allowance for the extracellular fluid contribution of MTX to the total, and the kinetic constants of the enzyme concerned.

This has been applied to the small intestine from C57BL mice treated with 400 mg/kg of MTX followed by rescue with either saline, thymidine, hypoxanthine + allopurinol, hypoxanthine + allopurinol + thymidine or folinic acid. In all the rescued groups, methotrexate levels in plasma, bone marrow and gut were higher than those in the saline control group. In all groups, inhibition of DHFR was greater than 95% for the duration of the rescue period (5 days). These results suggest that the survival of the rescued animals must depend upon an endogenous supply of reduced folates, or purines and pyrimidines, rather than recovery of DHFR activity in the gut.

**EFFECTOR REGULATION: A POTENTIAL NEW CHEMOTHERAPEUTIC STRATEGY.** R. M. PAINE and K. R. HARRAP, *Department of Biochemical Pharmacology, Institute of Cancer Research, Sutton, Surrey.*

At the last AGM we described how it was possible to enhance the toxicity of adenosine (AR) to lymphoid cells with coformycin (Cf), a tight binding inhibitor of adenosine deaminase (EC 3.5.4.4) (Harrap *et al.* (1976) *Br. J. Cancer*, **34**, 309). In the case of mitogenically-stimulated lymphocytes it was apparent that the biochemical mechanism underlying cytotoxicity was a build up of deoxyadenosine triphosphate (dATP) and an associated inhibition of DNA synthesis (see also Harrap and Paine (1977) *Adv. Enz. Regln*, **15**). In the present investigation, we have found that deoxyadenosine (AdR) is toxic to cultured L1210 cells in presence of deoxycoformycin (dCf). Giant-cell formation is detected by 24 h, due to inhibition of DNA synthesis in the face of continued RNA and protein synthesis (imbalanced growth). The primary metabolic lesion would appear to be inhibition of ribonucleotide reductase (EC 1.17.4.1) via a build up of the negative effector, dATP. Furthermore, treatment of L1210-tumour-bearing animals with binary combinations of AdR and dCf produces extension of survival time. We propose that selective enhancement of the regulatory properties of effector molecules provides a realistic means of inhibiting tumour growth.

**SOME KINETIC PARAMETERS OF PURINE SALVAGE ENZYMES.** D. C. TALBOT and K. R. HARRAP, *Department of Biochemical Pharmacology, Institute of Cancer Research, Sutton, Surrey.*

In view of the use of hypoxanthine (Hx) in selective methotrexate (MTX) rescue schedules (Straw *et al.* (1977) *J. natn. Cancer Inst.*, **58** 91; Talbot *et al.* (1976) *Br. J. Cancer*, **34**, 321), and the toxic effects of adenosine (AR) to normal and malignant lymphoid cells (Harrap *et al.* (1976) *Br. J. Cancer*, **34**, 321) it became important to understand the differences in purine salvage activity in a number of cell types. We have compared kinetic parameters of hypoxanthine guanine phosphoribosyl transferase (HGPRT) (EC 2.4.2.8) from L5178Y cells growing in purine-free ($Pu^-$) and purine-supplemented ($Pu^+$) medium. A 5-fold increase in $V_{max}$ of HGPRT from L5178Y ($Pu^+$) was observed 8 weeks after transferring cells into culture. In the L5178Y ($Pu^-$) line a 4-fold decrease in $V_{max}$ was found. The L5178Y ($Pu^-$) line had a greater rate of purine synthesis *de novo* than L5178Y ($Pu^+$). Little change in the $K_m$ for Hx was observed in HGPRT from either line. Selective potentiation of the toxic effects of AR by inhibitors of adenosine deaminase (ADA) (EC 3.5.4.4) depends on the relative rates of phosphorylation and deamination of AR. Adenosine kinase (AK) (EC 2.7.1.20) from mouse liver, spleen, marrow and L1210 cells had a greater affinity for AR than ADA, but the $V_{max}$ of ADA was ~100-fold greater than AK in these tissues. In L1210, the deamination/phosphorylation ratio increased markedly with AR concentration. This was not observed in spleen, liver or marrow. Studies have also indicated that AK from L1210 is substrate-inhibited at high concentration of AR. These studies show the importance of the kinetic parameters of purine salvage enzymes in relation to Hx rescue and AR toxicity.

**AN ASSESSMENT OF ORAL METHOTREXATE SYRUP.** J. G. McVIE, J. PAXTON, B. W. WHITING, M. SOUKOP and K. C. CALMAN, *Department of Clinical Oncology, Western Infirmary, Glasgow.*

A new formulation of methotrexate (MTX) was sought, to cope with the high doses in current practice. The standard tablet size is 2·5 mg, and so a syrup was prepared which had a final concentration of 2 mg MTX per ml. It was tested in the clinic and was accepted well by our patients. Samples of the syrup were assayed 8 times throughout 32 days of storage at room temperature or 4 °C by a radioimmunoassay for MTX. There was no alteration at all in the concentration of MTX throughout the experiment. Six patients had serial blood and urine samples taken after ingestion of the oral syrup at a dose of 50 $mg/m^2$ and then a week later after injection of an identical dose i.v. The relative availability of the drug varied from 15 to 62%. This indicated that absorption of the oral drug had taken place in all the patients, but the bio-available levels were considerably less in all cases than when the drug was given i.v. in the same dose. The mean $\gamma t_{1/2}$ in hours was 4·14 h for the i.v. route and 3·12 h by the oral route. There was marked individual variation in the handling of MTX in the 6 patients irrespective of the route of administration. We conclude that oral MTX may be given in a higher dose and possibly more frequently than i.v. MTX. Further, MTX syrup is stable over 32 days, and has proved extremely palatable to large numbers of patients.

**TREOSULFAN (DIHYDROXYBUSULPHAN) IN THE MANAGEMENT OF OVARIAN CARCINOMA.** J. J. FENNELLY, *St Vincent's Hospital, Dublin.*

Treosulfan (dihydroxybusulphan) is an alkylating agent which was synthesized by Feit in 1964. It has been found effective in Dunning Leukaemia and Lymphoma 8. Because of reports of Lundvall, Sorenson and Larsen (1973, *Acta obstet. Gynaec. Scand.* Suppl. 22) of benefit in ovarian carcinoma the author has evaluated Treosulfan in 40 patients with ovarian carcinoma. Treosulfan was given as 250-mg capsules q.i.d. daily for 4 weeks on alternate months. Depression of white cell count and platelet count occurred in similar pattern to that of other alkylating agents, but recovery was rapid. In addition a significant depression of haemoglobin occurred.

Of 40 patients treated, 12 (30%) showed a complete response for a mean duration of 15/12. Eleven (27%) showed a partial response for a mean of 7 months. 57% showed a total response.

Treosulfan is well tolerated, has a low level of gastrointestinal toxicity, is a predictable and transient marrow depressant, and gives response rates similar to other alkylating agents.

**COLCHICINE ULTRASENSITIVITY OF PERIPHERAL BLOOD LYMPHOCYTES IN LYMPHOID MALIGNANCIES.** J. H. SCARFFE, J. PRUDHOE and D. CROWTHER, *CRC Department of Medical Oncology, Christie Hospital and Holt Radium Institute, Manchester.*

The ultrasensitivity of chronic lymphatic leukaemia cells cultured for 20 h with colchicine, compared with normal lymphocytes, has been described by Thompson *et al.* (1972, *Scand. J. Haemat.*, 9, 231). We have used this technique to study peripheral blood lymphocytes in other lymphoid malignancies. Peripheral blood lymphocytes were incubated at 37 °C in 5% $CO_2$ for 20 h at concentrations of 0, $10^{-2}$, $10^{-3}$, $10^{-4}$, $10^{-5}$, $10^{-6}$, $10^{-7}$ M colchicine in TC199. The cells were wet fixed on slides, stained and the percentage of cells with pyknotic nuclei counted. Twenty normal controls all showed less than 20% pyknosis at all concentrations less than $10^{-2}$ M colchicine. Twenty-six ill controls, with diseases other than lymphoid malignancies, showed a slightly higher range of pyknosis up to 20% at concentrations less than $10^{-2}$ M. We were able to confirm the ultrasensitivity of chronic lymphatic leukaemia cells in 17/18 cases studied. Forty and 99% pyknosis at the lower concentrations of $10^{-7}$ and $10^{-6}$ were observed, compared with the concentration of $10^{-2}$ M required for pyknosis in normal lymphocytes. The one resistant case was initially sensitive but later developed resistance, although the absolute number of sensitive cells remained approximately the same. Peripheral blood lymphocytes in 17 cases of multiple myeloma were not found to be ultrasensitive. However 20/51 patients studied with non-Hodgkin lymphoma showed an abnormally high percentage of pyknotic cells. It was expected that ultrasensitivity would be found more commonly in the well differentiated pathology groups, but results were similar for both well and poorly differentiated groups. None of the patients studied was frankly leukaemic, all the abnormal group had lymphocyte counts less than 7000/$\mu$l.

**GROWTH KINETICS OF HUMAN TUMOUR XENOGRAFTS UPON SERIAL PASSAGE IN IMMUNE-DEPRIVED MICE.** J. A. HOUGHTON and D. M. Taylor, *Radiopharmacology Department, Institute of Cancer Research, Sutton, Surrey.*

Using the tumour systems previously described (Houghton and Taylor (1976) *Br. J. Cancer*, **34**, 313), preliminary studies have shown increased growth rates within the first 10 serial passages in 5/6 human colorectal tumours maintained in immune-deprived CBA/LAC mice. Using bilateral implants, the percentage of single tumour takes decreases significantly with serial passaging, with hosts producing either two or no tumours. These values deviate from those expected from a binomial distribution after the first one to two passages, more single takes being predicted than occur. Growth rates of individual tumours, calculated both at 0·4 $cm^3$ volume and during exponential growth, can differ widely on very early passages, and subsequently become more uniform after the first 2–4 passages. Volume-doubling times of tumours growing within the same animal are similar, and growth-rate variation within a passage is such that the variance of growth rates of tumours established in different mice is greater than those of tumours growing in the same animal. Hence fast and slow-growing tumours occur in separate hosts. Tumours which occur as single takes also have very varied growth rates within a passage. Results thus indicate a trend toward increased and more uniform growth rates. However, actual growth rates and takes per mouse within a passage may be dictated by the host. This could depend on the extent of individual host immune-deprivation which may influence the rate of cell loss within a tumour.

**CHANGES IN $^3$H-THYMIDINE UTILIZATION AS A PREDICTOR OF GROWTH DELAY IN FOUR HUMAN COLONIC TUMOUR XENOGRAFTS.** P. J. HOUGHTON, *Institute of Cancer Research, Sutton, Surrey.* (*Introduced by D. M. Taylor.*)

Changes in the fractional incorporation (FI) of radiolabelled precursors into DNA have been examined in 4 xenograft lines maintained in immune-deprived mice, and have been related to growth inhibition induced by the same treatment. The FI measures the proportion of the total radiolabelled precursor in the tissue sample which is incorporated into DNA within 1 h of administration. The FI is not affected by variation in precursor concentration achieved over a 10-fold range, which may occur in irregularly perfused tumours. Significant growth inhibition has been observed only when the cytotoxic agent produced a considerable and prolonged depression in $^3$H-thymidine FI. Tumour growth rate returns to its pretreatment value at a time when FI returns to the pretreatment level (FI recovery time). Following administration of cyclophosphamide, 5-fluouracil, or actinomycin D, the growth delay and FI recovery time are always similar. The initial depression of $^3$H-thymidine FI into DNA is a poor indicator of the actual growth delay, as different xenograft lines exhibiting the same depression 1 to 2 days after treatment may show considerably different FI recovery times, which are similar to the measured growth delay. However, within a tumour line there is a relationship between the initial depression of $^3$H-thymidine FI and both FI recovery time and growth delay, which appears to be independent of the mechanism by which the agent induces cell kill.

**CYCLOPHOSPHAMIDE AND *CIS*-DICHLORODIAMMINE PLATINUM (II): A PERSPECTIVE IN SCHEDULING.** K. D. TEW and D. M. TAYLOR, *Radiopharmacology Department, Institute of Cancer Research, Sutton.*

Fractional incorporation (FI) of $^3$H-thymidine (proportion of total tissue $^3$H incorporated into DNA) has been used successfully as a parameter for judging temporal scheduling of cyclophosphamide (CY) and *cis*-dichlorodiammine platinum (DDP). A difference in recovery time of FI following a dose of 100 mg/kg CY between tumour (>12 days), gut (2–3 days) and bone marrow (4 days) suggested a basis for a normal tissue-sparing drug regimen when administering double-dose CY-DDP therapy. There were 0/10 survivors when 100 mg/kg CY and 8 mg/kg DDP were administered together 1/10 survivors when the doses were separated by 1 day and 10/10 survivors when separated by 4 days. This 4-day interval was considered to allow gut and bone marrow recovery, factors crucial to the survival of the animal, before the second insult. These three combinations were similar in their antitumour effect in being slightly more than additive. CY was more myelotoxic than DDP; DDP was more gut-toxic. The recovery of bone-marrow cellularity was 2 days later than FI recovery. Peripheral white blood counts were reduced for a still longer period of time. The possibility of multiple drug administration based upon these findings remains to be elucidated.

**PHARMACOKINETICS OF PLATINUM ANTITUMOUR AGENTS IN MOUSE ORGANS.** B. W. MALERBI and G. ABEL, *Johnson Matthey Research Centre, Sonning Common, Oxon and Chester Beatty Research Institute, London.* (*Introduced by T. A. Connors.*)

In antitumour screening tests in BALB/C mice, *cis*-diamminedichloroplatinum (II) (DDP), *cis*-dichlorobis(cyclohexylamine) platinum (II) (CHP), and *cis*-dichlorobis (4-methylcyclohexylamine)platinum (II) (MCHP) gave $LD_{50}$ values of 16, 3200, and 1180 mg/kg. Corresponding $ID_{90}$ doses were 2·4, 12 and 990 mg/kg respectively. To elucidate these differences, these compounds were injected i.p. into healthy BALB/C mice. Animals were sacrificed at intervals spanning 15 min to 14 days after injection, and platinum analyses were performed on the liver, spleen, kidneys, heart, lungs, small intestine, large intestine, brain, skeletal muscle, bone and skin. Although all these compounds were stored in the liver, the effect was more marked for CHP and MCHP. In the kidneys, DDP produced an initial peak that declined rapidly, whereas CHP and MCHP produced lower steady levels. The large intestine showed a late rise in CHP and MCHP which was not observed with DDP. None of the compounds showed superior ability to cross the blood-brain barrier. The pharmacokinetic behaviour of CHP and MCHP in the kidney may explain their low toxicity compared with DDP. DDP is known to be excreted mainly via the kidneys, but the high levels of CHP and MCHP found in the intestines suggest that biliary excretion predominates for them. The differences in uptake are explicable by low aqueous solubility of CHP and MCHP, which persist in the peritoneum for several days. This slow uptake and sustained concentration in the tissues shown by CHP may explain its good antitumour activity. However, MCHP has similar pharmacokinetic behaviour but very low activity.

**THE MECHANISM OF INTERACTION OF TWO PLATINUM CO-ORDINATION COMPLEXES WITH RADIATION IN CHO CELLS *IN VITRO*.** A. H. W. NIAS and IRENA I. SZUMIEL,* *Glasgow Institute of Radiotherapeutics and Oncology, Belvidere Hospital, Glasgow.*

The effects of two platinum coordination complexes have been compared on CHO cells *in vitro*. While *cis*-dichlorobisisopropylamine trans-dihydroxy platinum IV (CHIP) is very soluble in water, *cis*-dichlorobiscyclopentylamine platinum II (PAD) is insoluble and was dissolved in DMSO. Dose-response curves after a 15 min exposure to CHIP and PAD were similar in shape, with final exponential slopes of 16 and 14 $\mu$g/ml respectively. The shoulder of the CHIP curve was much larger (N=300) than that for PAD (N=7).

Drug-radiation combination experiments showed a synergistic effect only when radiation followed a drug dose level high enough to reduce cell survival from the shoulder towards the exponential part of these dose-response curves. After PAD, the highest enhancement ratio was 1·59, whilst a ratio of 1·73 was found after a comparatively lower dose of CHIP. No cycle-phase specificity was found following PAD alone, but combinations with radiation showed more synergism in the $G_1$ and late-S position of the cell cycle than in mid-S.

These survival data, together with the results of other studies with PAD, including an absence of split-dose sparing and a pattern of chromatid aberrations, are compatible with the "molecular theory of cell survival" (Chadwick, Leenhouts, Szumiel and Nias (1976) *Int. J. Radiat. Biol.*, **30**, 511) which

* IAEA Fellow, Department of Radiobiology and Health Protection, Institute of Nuclear Research, 03-195, Warsaw, Poland.

provides an explanation of the cytotoxic action of the platinum complex and its synergistic interaction with radiation.

**THE EFFECT OF ICRF 159 ON ACCUMULATION AND REPAIR OF RADIATION DAMAGE.** I. W. TAYLOR and N. M. BLEEHEN, *MRC Unit of Clinical Oncology and Radiotherapeutics, Cambridge University Medical School.*

ICRF 159 has been shown to increase the sensitivity to X-irradiation of exponentially growing EMT6 mouse tumour cells *in vitro* (Taylor and Bleehen (1977) *Br. J. Cancer*, **36**). This is found only with ICRF 159 exposure times greater than 14 h, and only when the drug is given prior to irradiation. A 24-h exposure to 200 $\mu$g ICRF 159 before irradiation leads to a reduction in the radiation survival-curve shoulder (Dq=129 rad) compared with non-drug-treated controls (Dq=509 rad). This would suggest a loss of ability to accumulate or repair sub-lethal damage. The split-dose radiation response was examined using cells which had either a 6-h or a 24-h exposure to 200 $\mu$g ICRF 159 before the two doses of radiation and compared to control cells irradiated under similar conditions. In the ICRF 159-treated cells the drug was present during the interval between radiation doses. In all three cases, the cells, whether drug-treated or not, were found to have recovered 75–85% of their previously measured Dq. The reduction in Dq found for cells treated with ICRF 159 for 24 h, therefore, cannot be explained by the drug preventing or inhibiting *repair* of sub-lethal damage. It would appear therefore, that prolonged pretreatment with ICRF 159 reduces the cells ability to *accumulate* sub-lethal damage.

**STUDIES OF RESISTANCE TO ICRF 159 IN CELL LINE BS/159-1.** K. WHITE and A. M. CREIGHTON, *Imperial Cancer Research Fund, London.*

The isolation of a cell line (BS/159-1), derived from BHK 21S cells and showing resistance to the antitumour drug ICRF 159, has been previously reported (White and Creighton (1976) *Br. J. Cancer*, **34**, 323). Protein synthesis inhibitors (e.g. puromycin and cycloheximide) normally allow cells to progress into mitosis for 1 h only, after which time cells will no longer cross the $G_2$/M border. BS/159-1 cells, however, are not inhibited in this way, mitotic cells continue to accumulate. This suggests that either the protein requirement for mitosis is already met, or the protein-synthesis mechanisms in BS/159-1 cells are resistant to these inhibitors. The latter seems unlikely, since BHK 21S and BS/159-1 cells are equally sensitive to puromycin inhibition of protein synthesis. ICRF 159 has no direct effect on protein-synthesis mechanisms *per se*.

It is possible that ICRF 159 inhibits the function (either directly or indirectly) of a protein required for mitosis. The availability and/or nature of this protein may be modified in BS/159-1 cells.

### PART II: POSTER EXHIBITS

### ABSENCE OF NUCLEOSIDE EFFECT IN CELLS IRRADIATED BY FAST NEUTRONS.

A. Ferle-Vidović, D. Petrović, J. Sorić, D. Rendić and I. Šlaus, *Institute Ruder Boskovic, Zagreb, Yugoslavia.*

Breakdown products of DNA can increase the survival of irradiated cells. This had been studied extensively by employing deoxyribonucleosides in L cells (Petrović, Ferle-Vidović, Habazin, Vuković (1970) *Int. J. Radiat. Biol.*, **18**, 243) after X irradiation. In the present work, L 929 cells were irradiated by neutrons of different energies: 4–5 MeV mean energy and 14·5 MeV mono-energetic neutrons. For comparison, cells were also irradiated by $^{60}$Co gamma rays. Following irradiation cells were treated by an equimolar solution of deoxyribonucleosides (50 $\mu$g/ml), and effect on their survival measured. Results show that nucleoside treatment was efficient after the low LET irradiation: gamma rays survival curves were altered by nucleosides in terms of significantly increased extrapolation numbers only, but without $D_0$ change. Cells irradiated by neutrons from either of the two sources did not respond to nucleoside treatment, and consequently their survival curves remained unaltered. These results show that the nucleoside effect does occur after low LET irradiation, but apparently not following high LET irradiation. Since nucleosides as well as other cell breakdown products are released in irradiated tumours due to mass cell destruction, such nucleoside effect could possibly enhance the cell survival and thus affect the result of radiotherapy. Absence of the nucleoside effect in case of high LET irradiation may therefore be an additional potential gain from neutrons in radiotherapy.

### CELL CYCLE ANALYSIS *IN VITRO* USING FLOW CYTOFLUORIMETRIC TECHNIQUES.

J. V. Watson, *MRC Clinical Oncology and Radiotherapeutics Unit, Cambridge University Medical School.*

Flow cytofluorimetric technology enables the DNA content of cells in a single cell suspension to be estimated (Trujillo and van Dilla (1972) *Acta Cytol.*, **16**, 26). The first peak of a characteristic histogram corresponds to the fluorescence emitted by the DNA-fluorochrome complex of cells in $G_1$. The emission of cells in $G_2 + M$ is double that of cells in $G_1$ resulting in a second peak at double the abscissa scale reading, channel number. Two computer models are presented which can analyse the experimental data. The first employs age distribution theory (Steel (1968) *Cell & Tissue Kinet.*, **1**, 193) to give estimates of not only the proportions of cells in each phase, but also of the relative phase durations. This model can be used for populations containing a mixture of cycling and non-cycling cells, but it is concluded that reliable estimates of the growth fraction can only be obtained if the relative phase durations are known. Good agreement between the computed proportion in S phase and the $^3$H-TdR labelling index was found in the five cell lines analysed. A second model based upon the theory presented by Hartmann and Pederson ((1970) *Cell & Tissue Kinet.*, **3**, 1), has been produced to analyse the desynchronization of EMT6/M/CC cells following mitotic selection synchronization. Good agreement was obtained between the cytofluorimetric data and results from parallel $^3$H-TdR studies.

### STUDIES ON A DNA CONTAINING MATERIAL FROM P388 CELL LYSATES HIGHLY SENSITIVE TO IONIZING RADIATION.

D. G. Poppitt and B. W. Fox, *Paterson Laboratories, Christie Hospital and Holt Radium Institute, Manchester.*

The sedimentation behaviour of a DNA complex material resulting from lysis of P388 lymphoma cells following X- and gamma-irradiation has been studied on isokinetic sucrose gradients with an initial sucrose concentration of 20%. A decreased sedimentation rate following irradiation throughout the range from 5 rads to 10 krads has been observed. Post-irradiation incubation has suggested that a partial reconstitution of this material may occur within approximately 6 h.

**DEGRADATION OF ERROR PROTEINS IN HeLa CELLS.** D. N. Wheatley, M. R. Giddings, M. S. Inglis and J. H. Stevenson, *Department of Pathology, Aberdeen University Medical School.*

The possibility that changes in cell behaviour seen in phenomena such as ageing, malignant transformation, differentiation and mutation may be the result of error (or accumulation of errors) in protein biosynthesis (*e.g.* Orgel (1973) *Nature*, **243**, 441; Talmud and Lewis (1974) *Nature*, **249**, 563; Bradley and Schimke (1973) in *Intracellular Protein Turnover*, Academic Press, p. 311) is currently receiving much attention. The hypothesis raises the question of whether a special surveillance mechanism exists through which error proteins are detected and preferentially removed by cells, and the problems of what happens if it breaks down or is overloaded. In HeLa cells allowed to incorporate amino acid analogues instead of natural amino acids, we found no evidence of preferential degradation of anomalous proteins. In some cases, the consequences of analogue incorporation resulted in cells becoming degenerate with a subsequently elevated breakdown of all cellular proteins. The results favour the hypothesis that degradation follows first order kinetics for both normal and abnormal proteins and is due to a common intracellular proteolytic system operating in a stochastic manner.

**ACQUIRED DRUG RESISTANCE: ENHANCEMENT OF THE INTRANUCLEAR REACTIVITY OF ALKYLATING DRUGS.** R. Wilkinson and K. R. Harrap, *Department of Biochemical Pharmacology, Institute of Cancer Research, Sutton, Surrey.*

Alkylating agents find wide usefulness in the treatment of malignant diseases, though their effectiveness is impaired frequently by the development of acquired resistance. It becomes important therefore to devise schedules which, ideally, are antagonistic in terms of host toxicity and synergistic in relation to their antitumour effects. Alkylating agents are frequently administered in combination with steroids: we have found that binary combinations of chlorambucil and prednisolone can be administered to tumour-bearing (Yoshida sensitive and resistant) Wistar rats, producing a greater therapeutic index than can be achieved with chlorambucil alone. Similar results can be obtained with prednimustine (Leo 1031) a prednisolone ester of chlorambucil. Previous work in this laboratory has shown that chlorambucil induces morphological and chemical changes in the structure of nuclear proteins of drug-sensitive cells, though not of resistant cells (Riches and Harrap (1973) *Cancer Res.*, **33**, 389; Riches and Harrap (1975) *Chem.-Biol. Interactions*, **11**, 291). However, it will be shown that in the presence of prednisolone, similar changes can be produced in the chromatin of drug-resistant cells. We have also found that the time sequence of steroid administration, in relation to that of the alkylating agent, modifies the pattern of DNA cross-linking in the tumour, possibly by disruption of repair processes.

**MYELOTOXICITY OF METHOTREXATE IN ANIMALS WITH PYOGENIC INFECTION.** B. Harding and I. C. M. MacLennan, *Nuffield Department of Clinical Medicine, Radcliffe Infirmary, Oxford.*

This poster reports an investigation of the hypothesis that increased proliferative activity by neutrophil precursors induced by pyogenic infection will result in an increase in the susceptibility of these cells to damage by methotrexate (MTX). Rats were stimulated into prolonged increased neutrophil production by the induction of a unilateral pyohydronephrosis. Consistent profound neutropenia was seen when MTX was given during the first 2 days of infection, but thereafter greater neutropenia than that observed in non-infected rats was only observed in occasional animals. There was a significant correlation between the degree of neutropenia induced by MTX and the day after MTX upon which this occurred. It is argued that the main target for myelotoxicity by MTX is the myelocyte and that its precursors are relatively insensitive.

**MECHANISMS OF *IN VITRO* AND *IN VIVO* RAZOXANE RADIOSENSITIZATION.** M. Barker-Grimshaw, *Chemotherapy Department, Imperial Cancer Research Fund, London.* (*Introduced by Q. Hellman.*)

Razoxane (ICRF 159) produces radiosensitization *in vitro* and *in vivo*. Various mechanisms have been suggested to account for this effect, viz: the angiomorphic effect; blockage of cell cycle progression at $G_2/M$; inhibition of repair of radiation-induced DNA damage and general synergism with other antitumour agents, but which, if any of these mechanisms is involved is not yet clear. Experiments to test whether tumours treated with razoxane had an increased blood flow as a result of the angiometamorphic effect have been essentially negative. On the other hand, a higher oxygen concentration was found in such tumours even when treatment was delayed until 1 h before measurements were made. Compared with the inhibitory effect of razoxane or radiation alone, the combination of the two in the treatment of sarcoma S180 was much more effective even when razoxane was given 3 h after the radiation. Of the mechanisms of radiosensitization suggested for razoxane therefore that of inhibition of repair of radiation-induced DNA damage seems to be the most likely.

**FACTORS DETERMINING THE RESPONSE TO 5-FLUOROURACIL IN HUMAN COLONIC TUMOUR XENOGRAFTS.** P. J. HOUGHTON, J. A. HOUGHTON and D. M. TAYLOR, *Division of Radiopharmacology, Institute of Cancer Research, Sutton, Surrey.*

The relationship between the inhibition of $^3$H-deoxyuridine incorporation into DNA and growth delay following 5-fluorouracil administration has been examined in 4 human colonic tumour xenografts growing in immune-deprived mice. The dose of 5-fluorouracil producing 50% inhibition of $^3$H-deoxyuridine incorporation *in vivo* ($ID_{50}$) in 2 tumour lines was less than that found for normal (mouse) "limiting" tissues, but greater in the other tumour lines. After 5-fluorouracil (100 mg/Kg : $ID_{90}$ in all tumour lines) only tumours of one line showed a depression in $^3$H-thymidine incorporation into DNA and growth delay, whereas tumours of the other lines showed an increase in uptake and incorporation of this nucleoside into DNA during the first 4 days, and no growth delay. Recovery of $^3$H-deoxyuridine incorporation to the pretreatment level varied from 150 to over 600 h between tumour lines after 100 mg/Kg 5-fluorouracil. The difference between the recovery times for *de novo* ($^3$H-deoxyuridine) and "salvage" ($^3$H-thymidine) pathways after 5-fluorouracil treatment has been used as a measure of the ability of that human tumour line to utilise the "salvage" pathway for thymidine triphosphate synthesis in the presence of thymidylate synthetase inhibition. There appears to be no correlation between the degree or duration of thymidylate synthetase inhibition and growth delay, following 5-fluorouracil administration, in these xenografts. The ability of the tumour to use the "salvage" pathway for thymidine triphosphate synthesis appears to determine the response of these tumours in the presence of *de novo* thymidylate synthesis inhibition induced by 5-fluorouracil.

**THE PIG AS A MODEL FOR TOXICITY AND THERAPY TESTING OF CYTOTOXIC DRUGS.** S. E. BROWNLIE, J. G. CAMPBELL, K. W. HEAD, P. IMLAH, H. S. MCTAGGART and J. G. MCVIE, *Department of Clinical Oncology, Western Infirmary, Glasgow.*

A hereditary form of lymphoma associated with an autosomal recessive gene in Large White pigs is diagnosable before 3–4 months of age and fatal by about 15 months. The suitability of this condition for therapy testing of cytotoxic drugs has been investigated in toxicity tests using normal pigs. Prednisolone, dexamethasone, doxorubicin, cyclophosphamide and vincristine have been tested as single agents in normal and lymphomatous pigs. The results of treatment mimic those expected in humans suggesting that this is a good animal model for testing new drugs and novel schedules of established drugs. Prednisolone and dexamethasone used separately produced an increase in serum albumin and marked involution of the thymus with "overshoot" on withdrawal of drugs in both normal and lymphoma pigs. A similar effect has been reported in human infants (Caffey and Silbey (1960) *Paediatrics*, **26**, 762). A striking reduction in circulating lymphocytes and in the size of lymph nodes occurred particularly in the lymphoma cases. Beneficial effects were observed in red cell picture, neutrophil and platelet counts and on the general vigour and well-being of lymphoma cases. These effects were all more marked with prednisolone than with dexamethasone. As in man, doxorubicin was cumulatively toxic at high doses in both normal and lymphoma pigs, producing stunting of growth, buccal ulceration, alopecia, diarrhoea, liver damage, leucopenia, thrombo-cytopenia and cardiotoxicity. Remission has been achieved and maintained for over a year in one case given cyclophosphamide and vincristine in combination with steroids.

**CANINE OSTEOSARCOMA: COMBINATION CHEMOTHERAPY AND CLINICAL STAGING.** A. M. HENNESS, *Clinical Oncology Service, University of California, U.S.A.* (*Introduced by L. N. Owen.*)

There are few reports on the use of antineoplastic drugs in therapy for canine osteosarcoma (OS); results with these agents have not been encouraging, either because of their lack of effectiveness against the disease or due to their significant toxicity to the host. In this initial study of use of combination chemotherapy for canine OS, 11 dogs were given cytotoxic drugs following amputation. Drugs in the standardized 6 month protocol were: Adriamycin (30 mg/$m^2$), cyclophosphamide (50 mg/$m^2$), and methotrexate (5 mg/$m^2$) with citrovorum factor "rescue". A method of clinical staging of canine OS was devised, based on clinical and radiographic findings at time of diagnosis, to assist retroactively in the evaluating of therapy and survival data of these 11 animals. Their distribution by clinical stage was: 1, IIa; 4 IIIa; 5, IIIb; and 1, IVb. At 8 months from time of diagnosis, 7 (64%) were alive, and 5 (45%) were clinically free of metastases. One (initially stage IIIb) survived 23 months and then died suddenly without evidence of OS. A second (stage IIa) is alive at greater than 41 months from time of diagnosis. Drugs in the protocol appeared to be well tolerated.

**ANALYSIS OF TWO LYMPHOCYTIC LAYERS ACHIEVED BY FICOLL-TRIOSIL GRADIENT SEPARATION.** C. R. PENTYCROSS, *Department of Medical Oncology, Charing Cross Hospital, London.* (*Introduced by K. D. Bagshawe.*)

During studies on the structuredness of cytoplasmic matrix (SCM) of lymphocytes, 2 separate interface layers were sometimes obtained with a modified Ficoll-Triosil gradient separation technique described by Cercek and Cercek. Two layers were obtained with 6/10 normal bloods and 4/10 samples from cancer patients. The two layers have been compared. In both normal and cancer samples the upper layers contained from 92–97% lymphocytes; the lower layers were more contaminated with non-lymphocytic white cells. T (thymus-derived) cells were more predominant in the upper than lower in both groups. The SCM test in 8/10 normal subjects and 7/10 cancer patients confirmed results originally reported by Cercek *et al.* ((1974) *Br. J. Cancer*, **29**, 345), as regards the upper layers, i.e. more response to phytohaemagglutinin (PHA) than to myelin basic protein (MBP) in normals and the reverse in cancer patients. In 2/10 normal subjects and 3/10 cancer patients the lymphocytes were non-responsive to either substance. The lower layers, in both groups, were studied where yield permitted, but produced inconclusive SCM results. In 2 normal subjects, where the yield permitted study of PHA blastogenesis, the upper layers showed more transformation. It is concluded that the upper layers in all subjects studied contain a greater proportion of T lymphocytes, are less contaminated by other white cells, are more responsive to PHA stimulation in culture and give more definitive responses in the SCM test.

**SEPARATION OF HUMAN LYMPHOCYTES FORMING MOUSE RED CELL ROSETTES.** M. R. POTTER, *Paterson Laboratories, Christie Hospital and Holt Radium Institute, Manchester.* (*Introduced by M. Moore.*)

Subpopulations of lymphocytes with receptors for heterologous erythrocytes can be identified by rosetting tests as exemplified by the formation, by human T lymphocytes, of rosettes with sheep red blood cells (SRBC). Rosette formation between human lymphocytes and mouse red blood cells (MRBC) has been described more recently as a marker for B lymphocytes, or a subpopulation of B lymphocytes (Stathopoulos and Elliot (1974) *Lancet*, **i**, 600). MRBC rosette formation with human blood lymphocytes and the separation of rosette forming cells as a method of B lymphocyte enrichment has been examined. Blood lymphocytes were prepared by Ficoll-

Triosil gradient centrifugation and a small percentage of these cells (mean value 6%) formed spontaneous rosettes with MRBC under conditions similar to those used for SRBC rosette formation. The proportion of MRBC rosettes was increased (mean value 16%) by treating the lymphocytes with neuraminidase before rosetting. Neuraminidase treatment of the MRBC also increased the number of rosettes formed, but to a lesser extent (mean 11%). Double marker tests demonstrated that lymphocytes forming MRBC rosettes were immunoglobulin (Ig) bearing cells, with a high proportion of IgM bearing cells, but not all Ig bearing cells formed rosettes. Depletion of the proportion of B lymphocytes in the population by nylon fibre column filtration produced a corresponding fall in the number of MRBC rosette forming cells. Separation of rosette forming cells by Ficoll-Triosil gradient centrifugation gave a pellet population enriched for B lymphocytes and an interface population enriched for T lymphocytes. Tests on the degree of enrichment by re-rosetting with MRBC produced variable results whereas testing by SRBC rosette formation showed a consistent pattern of enrichment.

**T AND B CELL POPULATIONS IN CANCER PATIENTS AND CONTROLS USING FRESH AND FROZEN LYMPHOCYTES.** C. H. J. Ford, C. E. Newman and A. B. Carter, *University Department of Surgery, Queen Elizabeth Hospital, Birmingham.*

Individual E and EAC′ rosette tests and a combination assay for measuring E, EAC′ and mixed rosettes have been used to measure the numbers of T and B cells in the peripheral blood of 96 blood transfusion donors (BT), 15 laboratory staff (LS), 36 pre-operative patients with non-malignant surgical conditions (SP) and 40 cancer patients (CP). Statistically significant differences were obtained when comparing the three control groups (BT, LS, SP) with the CP group in the individual E test, $P < 0{\cdot}002$ ($74{\cdot}7 \pm 9{\cdot}7$, $79{\cdot}4 \pm 8{\cdot}7$, $72{\cdot}9 \pm 10$ vs $66{\cdot}2 \pm 15{\cdot}5$), combination E test, $<0{\cdot}02$ to $<0{\cdot}002$ ($71{\cdot}8 \pm 8{\cdot}9$, $73{\cdot}1 \pm 6$, $71{\cdot}9 \pm 8{\cdot}8$ vs $66{\cdot}1 \pm 10{\cdot}7$), and when comparing the BT and CP groups in the individual EAC′ test, $P <$ p·01 ($11{\cdot}35 \pm 4{\cdot}3$ vs $8{\cdot}9 \pm 4{\cdot}8$). A significant effect of freezing on rosetting ability in both tests was seen for E rosettes in the SP group ($P < 0{\cdot}002$) and for EAC′ rosettes in the BT group ($P < 0{\cdot}02$). In the CP group the combination EAC′ result was also significantly reduced ($P < 0{\cdot}01$). The possibility that the difference found between the CP and control groups is due to an effect of longer storage of blood from cancer patients prior to testing is being investigated.

**CLINICAL CORRELATES OF *IN VITRO* LYMPHOID FUNCTION.** N. Thatcher, N. Gasiunas and D. Crowther, *CRC Department of Medical Oncology, Christie Hospital and Holt Radium Institute, Manchester.*

Lymphoid function was investigated using the peripheral lymphocyte count, E, EAC rosettes and also direct, antibody dependent and PHA induced lymphocytotoxicity against $^{51}$Cr labelled Chang cells.

(a) Influence of Pathology and Stage in non-Hodgkin lymphoma. Thirty untreated patients were examined. Peripheral lymphocytes, antibody dependent cytotoxicity and E rosettes were reduced ($P < 0{\cdot}5$) compared with controls. The reduction was statistically significant for patients with diffuse pathology. Patients with nodular pathology, early and late stage disease, showed some reduction in test values but were not statistically significant.

(b) Influence of Surgical Removal of Primary Hypernephroma. Eighteen patients with untreated hypernephroma had significantly reduced antibody dependent ($P < 0{\cdot}01$) and PHA induced cytotoxicity ($P < 0{\cdot}02$) compared with the same patients 14 days post-nephrectomy or with normal controls. Seven patients had demonstrable distant metastases pre-operatively, these patients demonstrated a smaller post-operative rise in cytotoxicity than the 11 non-metastatic patients rendered clinically tumour free.

(c) Influence of Chemotherapy and Reduction in Metastatic Burden. Nineteen patients with metastatic head and neck, gastric and bladder carcinoma were treated with pulsed courses of Adriamycin and 5-Fluorouracil. The patients were immuno-suppressed ($P < 0{\cdot}02$) as indicated by peripheral lymphocyte count, E, EAC rosettes, direct and antibody dependent lymphocytotoxicity. Therapy which induced an objective reduction in metastases reduced this pretreatment immunosuppression, but non-responding patients showed further immunosuppression. Lymphoid function as measured by these methods is therefore related to clinical parameters of tumour type and tumour burden.

### HISTOCHEMICAL DETECTION OF ABNORMAL SACCHARIDES IN A CHONDROSARCOMA.

R. W. STODDART, D. D. DZIEWIATKOWSKY and S. FITTON-JACKSON, *Strangeways Research Laboratory, Cambridge.*

The Schwarm chondrosarcoma of the rat, which originated as a spontaneous osteochondrosarcoma, produces a matrix which is abnormally soluble in chaotropic agents and lacks the repeating sequence of keratan sulphate. It was maintained by subcutaneous and intraperitoneal transplantation in hooded rats and large tumours and occasional metastases were obtained. Samples were fixed in anhydrous methanol or Zenker-acetic acid and paraffin sections were made by conventional procedures. These were specifically stained for various sugars by fluorescent-labelled lectins. Comparisons were made with several cartilages of foetal, juvenile and adult rats. The malignant chondrocytes showed peculiarities of their nuclear and surface sugars. The matrix was highly abnormal and disordered. Soy-bean agglutinin stained "cable-like" structures running through it, which were not seen in any normal cartilage and which may have represented the unsubstituted residues of N-acetylgalactosamine by which keratan sulphate is normally linked to polypeptide. Concanavalin A stained glycogen intensely and detected fine fluffy fibrils in the matrix that may be disordered collagen.

### ATHYMIC NUDE MICE: HUSBANDRY AND TRANSPLANTATION STUDIES.

D. R. MORGAN, *Department of Clinical Veterinary Medicine, University of Cambridge.*

A breeding colony of nude mice was established using homozygous (nu/nu) males and heterozygous (nu/+) females, maintained under simple barrier conditions in isolation from other animals. Cages, sawdust, food and water were sterilized and maintenance personnel wore surgical gowns, hats and masks, and the animals were handled using sterilized gloves. The room temperature was held at 25–27 °C and the humidity (uncontrolled) was about 45%. The breeding colony consisted of permanently caged pairs and trios. Productivity was similar to previous reports (Festing and King, 1974) and although some perinatal mortality was in evidence a mean number of 3·5 nu/nu per litter at weaning was obtained. In most cases post-partum matings occurred and the young were delivered at about the time of weaning the previous litter. Mortality rates for both the breeding colony and experimental mice were low. Some cases of the common wasting-disease syndrome and skin abscesses were found together with occasional instances of rectal prolapse. The colony has been maintained for more than 3 years and the average life-span of homozygotes approached 9–12 months under these conditions. Neoplastic tissues from canine melanoma, osteosarcoma, mammary carcinoma and lymphosarcoma were made into cell suspensions and cultured in RPMI 1640 containing 10% FCS. Cells from 10 cultures were injected subcutaneously in duplicate ($5 \times 10^7$ cells) into 6–8 week old mice. Transplantation was successful in 7 cases (14 mice) and growth was assessed by serial measurement of the palpable tumours. Tumour samples were examined histologically and by electron microscopy and were re-cultured *in vitro.*

### THE AETIOLOGY OF BREAST CANCER AND THE OESTROGENIC METABOLITES OF FUSARIA.

R. SCHOENTAL, *Department of Pathology, Royal Veterinary College, University of London.*

Reviewing this subject and the many factors suspected as the causative agents, MacMahon, Cole and Brown ((1973) *J. natn. Cancer Inst.*, **50**, 21), concluded that the "nature of the familial factors, genetic or environmental, is unknown". I suggest that possible aetiological factors of breast cancer, which not yet have been taken into consideration, are the non-steroidal oestrogenic secondary metabolites of microorganisms (mainly of *Fusarium spp.*) such as zearalenone and its congeners, which can be found in some batches of stored grains (compare Stoloff (1976) in *Mycotoxins and other Fungi related*

*Food Problems*, Adv. Chem. Series, **149**, 23; Hacking, Rosser and Dervish ((1976) *Ann. Appl. Biol.*, **84**, 7).

The presence in human foodstuffs of oestrogenic substances, active by the oral route could explain the occurrence of familial breast cancer. Members of a family usually partake from the same food, hence would be similarly exposed to its contaminants. It is worth noting, that the presence of oestrogenic substances is not likely to be detected by taste, or by toxic effects following soon after ingestion. They act insidiously.

Specimens will be shown of Fusarium-infected maize containing zearalenone, as well as cultures of *Fusarium graminearum* on various media kindly supplied by P. K. C. Austwick, Nuffield Institute of Comparative Medicine, The Zoological Society of London, and of zearalenone-containing barley obtained from A. Hacking, Ministry of Agriculture, Fisheries and Food, Shardlow Hall, Shardlow, Derby.

### ISOLATION OF EPITHELIAL SHEETS OF HUMAN MAMMARY TUMOUR CELLS.

A. Howell, G. K. Panda and N. Ahktar, *Departments of Medicine and Cancer Studies, Birmingham University.*

The mean cell yield from 37 human mammary tumours using the collagenase di-aggregation technique of Lasfargues (J. Fogh (ed), *Human Tumour Cells in vitro*, Plenum Press, 1975) was $3{\cdot}90 \times 10^7$ cells/g wet weight with a mean viability of 55·6%. As judged by morphology lymphocytes comprised 17·8% of the cell population on average. The presence of lymphocytes was confirmed by rosetting: all tumours tested contained cells with Fc and C3 receptors and also cells which formed E rosettes. Latex ingesting cells formed approximately 1% of the total population. Adequate tumour cell culture was not obtained possibly because of the presence of lymphocytes initially and certainly because of fibroblast overgrowth later. Cells spilled at the time of cutting of tumours gave a mean yield of $2{\cdot}42 \times 10^7$/g original tumour wet weight and a mean viability of 17·3%. The high proportion of dead cells interfered with tumour cell aggregation and adhesion to the culture surface. When dead cells were removed by centrifugation through Ficoll-Triosil the viability increased to a mean of 74·5% and tumour cells were the predominant cell type. Viable cells aggregated while on the Ficoll, and these balls of tumour cells readily adhered to and spread over the culture surface to form epithelial islands and sheets with virtually no fibroblast contamination.

### THE ANTI-TUMOUR ACTION OF SENSITIZED PIG LYMPH NODE CELLS, MEASURED BY REDUCTION OF PULMONARY METASTASES IN MICE: OBSERVATIONS ON THE NECESSARY SPECIFICITY OF SENSITIZATION.

S. Prichard-Thomas and M. O. Symes, *Department of Surgery, University of Bristol.*

Pulmonary tumour metastases were induced in A-strain mice by i.v. injection of $10^6$ A-strain mammary carcinoma cells. In some mice, a splenectomy alone was performed on Day 6. Other mice received, in addition, on Day 7 an i.v. injection of $2 \times 10^7$ unsensitized or sensitized mononuclear cells, separated from the mesenteric lymph node chain of a pig. The mice were killed on Day 14, their lungs fixed in Bouin's fluid, and the number of metastases counted. The effect of splenectomy alone, and of splenectomy plus pig cells, in reducing the number of metastases, was assessed in comparison with the numbers in untreated mice. The results, obtained by an analysis of variance using pooled data from a number of experiments, were as follows:

*Series I*

| Treatment | No. of observations | Significance in reduction of pulmonary metastasis number |
|---|---|---|
| Nil | 27 | |
| Splenectomy | 18 | $<0{\cdot}05$ |
| Splenectomy + pig cells (unsensitized) | 10 | NS |
| Splenectomy + pig cells (sensitized to mouse tumour) | 23 | $<0{\cdot}01$ |

*Series II*

| Treatment | No. of observations | Significance in reduction of pulmonary metastasis number |
|---|---|---|
| Nil | 42 | |
| Splenectomy | 27 | NS |
| Splenectomy + pig cells sensitized to mouse tumour | 34 | $<0{\cdot}01$ |
| Sensitized to mouse skin | 17 (26)* | NS |
| Sensitized to human tumour | 20 (28)* | $<0{\cdot}05$ |

* No. of "nil" treatments for this comparison.

Thus only pig cells sensitized against the mouse tumour to be treated had a significant anti-tumour effect. Cells sensitized against a human tumour (Series II) were only marginally effective, producing the same degree of reduction in nodule number as splenectomy alone (Series I). Furthermore, when these experiments were repeated using pig mononuclear cells stimulated *in vitro* by culture for 2 days in the presence of PHA, no anti-tumour effect was obtained. The requirement for a population of pig cells *sensitized* against the tumour to be treated to obtain an anti-tumour effect, suggests that the mechanism thereof is an adoptive transfer of immunity from pig to mouse.

**MONONUCLEAR PHAGOCYTE PROLIFERATION IN INFLAMMATION AND *IN VITRO*.** K. M. WYNNE and W. G. SPECTOR, *Department of Pathology, St Bartholomew's Hospital Medical College, London. (Introduced by M. Moore.)*

Mononuclear phagocyte, or macrophage, proliferation is a well-established feature of the chronic inflammatory response, although its influence upon macrophage activation, and hence its exact contribution to lesion progression and/or resolution, remain to be elucidated. Cultured macrophages, in marked contrast to their inflammatory counterparts *in vivo*, exhibit only minimal levels of DNA synthesis and proliferation, a fact which can be exploited in the design of a model system to investigate the possible existence of local humoral mitogenic factors in inflammatory lesions. Exposure of *in vitro* macrophage monolayers to cell-free inflammatory exudate harvested from 4-day-old chronic lesions, resulted in a stimulation of DNA synthesis, as evidenced by $^3$[H]TdR incorporation and subsequent cell division. No response was observed prior to the 4th day, but by 7 days mean $^3$[H]TdR incorporation by exudate-treated cells had risen to 60% (range 46–83%) as compared to 1% (range 0–2%) in the case of control cells, and direct counting techniques indicated that exudate-treated, but not control, cell populations doubled in number between the 7th and 10th day of culture. Further prolongation of the proliferative response was not observed, under the culture conditions employed in the present study. Additional evidence for the existence of macrophage mitogenic activity in inflammatory exudate comes from the work of Adolphe's group in Paris (Adolphe *et al.* (1975) *Nature*, **253**, 637), and other studies have implicated both fibroblasts (Cifone *et al.* (1975) *Expl Cell Res.*, **96**, 96) and lymphocytes (Hadden *et al.* (1975) *Nature*, **257**, 483) as potential sources of such factors; thereby serving to emphasize the probable complexity of the cellular interrelationships and control mechanisms which exist in chronic inflammation.

**TARGET CELLS OF THE LEUKAEMOGENS BUTYL AND METHYL NITROSOUREA.** P. BAINES, *Paterson Laboratories, Christie Hospital and Holt Radium Institute, Manchester. (Introduced by M. Moore.)*

Lymphoblastic leukaemia developing in intact mice treated with a single i.v. dose of MNU or a chronic oral administration of BNU usually present with thymoma. The spleen, lymph nodes and liver may also be involved. Occasionally the leukaemia may develop in the spleen or lymph nodes in the absence of thymic enlargement. Thymectomy before MNU treatment greatly increases the induction time and decreases the incidence of leukaemias. The incidence of leukaemias in thymectomized BNU-treated mice depends upon the dosing regime. The incidence decreases and induction time increases if the dose duration is reduced from continuous feed to 4 weeks. A similar dose reduction has little effect on the characteristics of leukaemias developing in intact mice. A study of surface markers on terminal leukaemic cells shows that in mice with thymoma involvement $\theta^+$ve, Ig$^-$ve leukaemias result. If no thymus enlargement is observed the leukaemic cells are $\theta^+$ve, Ig$^-$ve, or $\theta^-$ve Ig$^-$ve. In thymectomized mice $\theta^-$ve, Ig$^-$ve or Ig$^+$ve leukaemias may develop. The leukaemias arising in intact and thymectomized mice may be derived from the same or different target cells. In order to investigate these possibilities, neonatal thymuses were grafted into thymectomized MNU- or BNU-treated mice. In most cases the grafted thymus did not bring about expression of $\theta$ antigen on terminal leukaemic cells. The thymus may be either the site of a target cell population or the site required by the target cell population for expression of $\theta$ and rapid proliferation. Thus thymectomy may remove either the target cell or essential factors required for maturation of the target cell. Leukaemias develop in thymectomized hosts, grafted with neonatal thymus, only after a prolonged induction time and only rarely express $\theta$ antigen. These results are consistent with there being a target cell population within the thymus.

**IMMUNOADSORBENT PURIFICATION OF A RAT SARCOMA SPECIFIC ANTIGEN.** V. E. Preston and M. R. Price, *Cancer Research Campaign Laboratories, University of Nottingham.*

Soluble fractions retaining tumour specific antigenic activity were prepared from a 3-methylcholanthrene-induced rat sarcoma by 3 M KCl treatment of tumour tissue. These extracts, being initially highly heterogenous, were fractionated by immunoadsorbent procedures involving: (a) the binding of tumour-specific antigen to syngeneic rat anti-sarcoma antibodies immobilized upon Sepharose 4B (Pharmacia, Uppsala), (b) elution of bound material with 3 M NaSCN followed by rapid desalting of the antigenic protein upon Sephadex G25, and (c) passage of the antigenic protein fraction over an immunoadsorbent containing normal rat serum IgG to remove contaminants which non-specifically bind to substituted Sepharose matrices. The material so obtained was characterized by its capacity to neutralize syngenic tumour specific antibody and to induce the formation of specific antibody in immunized rats. This antigenic fraction was further employed in the preparation of rabbit antisera which, following absorption with unrelated rat sarcoma cells, were rendered monospecific for the immunizing sarcoma. Although the material isolated showed limited heterogeneity as judged by polyacrylamide gel electrophoresis and gel filtration chromatography, the preparative procedure does allow the rapid recovery of fractions which are suitable for further separation and characterization. Also, the availability of these semi-purified antigen preparations as well as monospecific heteroantisera may aid the development of a quantitative radioimmunoassay for tumour specific antigens associated with chemically induced rat tumours.

**EXAMINATION OF THE MEMBRANE PROTEINS OF HUMAN MELANOMA CELL LINES.** G. P. Roberts, R. H. Whitehead and L. E. Hughes, *University Department of Surgery, Welsh National School of Medicine, Cardiff.*

Cell-surface components play an important role in regulation of cell growth, antigenicity and cellular recognition. There is increasing evidence that the plasma membranes of tumour cells differ from those of normal cells, but these studies have been largely confined to laboratory animal cells. Therefore, a study has been made of the cell surface proteins of human melanoma cell lines. The cell-surface proteins were labelled with $^{125}I$ or $^{131}I$ in the presence of lactoperoxidase, and the labelled proteins examined by SDS electrophoresis on a 5–22·5% acrylamide gradient followed by autoradiography. As many as 24 cell-surface proteins were detected in the individual melanoma cell lines; the molecular weights of the mercaptoethanol-reduced subunits ranged from about 10,000 to 240,000. There were considerable differences in the protein profiles of the different melanoma cell lines, only 10 of the proteins being common to all 4 melanoma cell lines examined, and 7 of these proteins were also detected on the cell surface of fibroblast cell lines. Attempts were made to detect melanoma-specific antigens by affinity chromatography of extracts of the labelled cells on immunoadsorbent columns prepared with an antisera against melanoma cells. Electrophoretic examination of the proteins bound by the immunoadsorbent columns did not reveal any proteins common to all melanoma cell lines but absent from other cell lines.

**HISTOLOGICAL AND IMMUNOLOGICAL RESPONSES IN THE DRAINING LYMPH NODE DURING TUMOUR GROWTH IN RATS.** G. Robinson,* J. A. Jones and R. C. Rees, **Department of Pathology and Cancer Research Campaign Laboratories, University of Nottingham.*

Little detailed information exists regarding the response of the draining lymph node to a developing tumour. Using a transplantable rat hepatoma (D192A) as a model, the histological and immunological changes of lymph nodes regional and distal to the tumour site were studied at various stages following tumour implantation into the hind limb. Histologically, an early cell-mediated response was detected in the lumbar node draining the tumour site. The T-dependent paracortex showed a marked proliferation of cells, along with increased numbers and prominence of post-capillary venules. This response was maintained for the first 3 weeks of tumour growth, and then the paracortex became depleted of lymphocytes. Stimulation of the cortical lymph follicles, with development of active germinal centres and migration of plasma cells to the medullary cords, was evident 11 days after inoculation, and this humoral response showed no signs of later inactivation. Similar morphological evidence of cell- and humoral-mediated responses to tumour growth was observed in distal nodes, but these were out of phase with those shown by the draining node. The response of the draining and distal lymph nodes was monitored using the *in vitro* microcytotoxicity test. Cells from the lumbar nodes displayed an early cytotoxicity against D192A and 15-day-old-embryo cell targets, which decreased during tumour growth. Cells from the cervical lymph nodes showed an increasing cytotoxic response towards these cell targets. In addition, the presence of serum antibody, specific for the developing tumour, was detected during the latter stages of tumour growth by indirect membrane immunofluorescence.